# Genome-wide SNP Genotyping Resolves Signatures of Selection and Tetrasomic Recombination in Peanut

**DOI:** 10.1016/j.molp.2016.11.015

**Published:** 2017-02-13

**Authors:** Josh Clevenger, Ye Chu, Carolina Chavarro, Gaurav Agarwal, David J. Bertioli, Soraya C.M. Leal-Bertioli, Manish K. Pandey, Justin Vaughn, Brian Abernathy, Noelle A. Barkley, Ran Hovav, Mark Burow, Spurthi N. Nayak, Annapurna Chitikineni, Thomas G. Isleib, C. Corley Holbrook, Scott A. Jackson, Rajeev K. Varshney, Peggy Ozias-Akins

**Affiliations:** 1 Department of Horticulture and Institute of Plant Breeding, Genetics & Genomics, The University of Georgia, 2356 Rainwater Road, Tifton, GA 31793, USA; 2 Center for Applied Genetic Technologies and Institute of Plant Breeding, Genetics & Genomics, The University of Georgia, Athens, GA 30602, USA; 3 International Crops Research Institute for the Semi-Arid Tropics (ICRISAT), Hyderabad 502324, India; 4 University of Brasília, Institute of Biological Sciences, Campus Darcy Ribeiro, 70910-900 Brasília, DF, Brazil; 5 Embrapa Genetic Resources and Biotechnology, 70770-917 Brasília, DF, Brazil; 6 USDA-ARS PGRCU, Griffin, GA 30223, USA; 7 Agricultural Research Organization, Plant Sciences Institute, 7528809 Rishon LeZion, Israel; 8 Department of Plant and Soil Science, Texas Tech University, Lubbock, TX 79409-2122, USA; 9 Department of Crop and Soil Sciences, North Carolina State University, Box 7629, Raleigh, NC 28695-7629, USA; 10 USDA-ARS, Tifton, GA 31793, USA

**Keywords:** single nucleotide polymorphism, groundnut, *Arachis hypogaea*

## Abstract

Peanut (*Arachis hypogaea*; 2n = 4x = 40) is a nutritious food and a good source of vitamins, minerals, and healthy fats. Expansion of genetic and genomic resources for genetic enhancement of cultivated peanut has gained momentum from the sequenced genomes of the diploid ancestors of cultivated peanut. To facilitate high-throughput genotyping of *Arachis* species, 20 genotypes were re-sequenced and genome-wide single nucleotide polymorphisms (SNPs) were selected to develop a large-scale SNP genotyping array. For flexibility in genotyping applications, SNPs polymorphic between tetraploid and diploid species were included for use in cultivated and interspecific populations. A set of 384 accessions was used to test the array resulting in 54 564 markers that produced high-quality polymorphic clusters between diploid species, 47 116 polymorphic markers between cultivated and interspecific hybrids, and 15 897 polymorphic markers within *A. hypogaea* germplasm. An additional 1193 markers were identified that illuminated genomic regions exhibiting tetrasomic recombination. Furthermore, a set of elite cultivars that make up the pedigree of US runner germplasm were genotyped and used to identify genomic regions that have undergone positive selection. These observations provide key insights on the inclusion of new genetic diversity in cultivated peanut and will inform the development of high-resolution mapping populations. Due to its efficiency, scope, and flexibility, the newly developed SNP array will be very useful for further genetic and breeding applications in *Arachis*.

## Teaser

Genome-wide single nucleotide polymorphisms (SNPs), identified among diploid and tetraploid Arachis species and used to construct an Affymetrix 60K SNP array, provided insight into diversity across US runner-type peanut cultivars and the US mini - core collection. The frequency of fixed marker polymorphisms and identification of preferentially selected haplotypes will inform future peanut breeding to enhance diversity.

## Introduction

Peanut (*Arachis hypogaea*; also known as groundnut) is an especially important nutritional resource for the poor and malnourished. With the advent of the genome sequences from the ancestors of peanut *A. duranensis* and *A. ipaensis*, peanut now has become “the orphan legume whose time has come” (Ozias-Akins, 2013, Bertioli et al., 2016). A beneficiary of serendipity, the accession of *A. ipaensis* Krapov. & W.C. Greg. collected and sequenced is probably from the same population that formed the hybrid with *A. duranensis* Krapov. & W.C. Greg. to become *A. hypogaea*. Genetic mapping studies in peanut have relied largely on SSR markers (Gautami et al., 2012, Qin et al., 2012, Sujay et al., 2012, Shirasawa et al., 2013, Huang et al., 2015), which are limited in number, time consuming, and expensive to assay. The use of high-throughput markers like those based on single nucleotide polymorphisms (SNPs) is needed for efficient application of genomics data for marker-assisted breeding, quantitative trait locus (QTL) mapping, and genomic selection.

Although SNP arrays have been used in *Arachis* crosses involving wild species (Nagy et al., 2012, Bertioli et al., 2014), the development of arrays that suitably assay the tetraploid (2n = 4x = 40) cultivated peanut (*A. hypogaea*) has lagged behind other polyploids. Polyploid species for which high-density SNP arrays have already been developed include cotton (Hulse-Kemp et al., 2015), oat (Tinker et al., 2014), wheat (Wang et al., 2014), and strawberry (Bassil et al., 2015). Although SNP validation rates were lower for these SNP arrays when compared with diploids, they still achieved success rates above 61% (Tinker et al., 2014, Wang et al., 2014, Hulse-Kemp et al., 2015); however, the overall success rate varied across SNP identification strategies in cotton (Hulse-Kemp et al., 2015). Gene-enriched sequence-supplied SNPs (RNA-sequencing data; gene-enrichment restriction libraries) had a higher success rate than genomic re-sequencing data-supplied SNPs (87% versus 49%), and genomic SNPs identified between species had a higher success rate than SNPs identified within species (59% versus 49%) (Hulse-Kemp et al., 2015). Cotton relied on the ample genetic diversity within polyploid germplasm, with five species of allopolyploid *Gossypium*, including three wild species and two cultivated (*G. hirsutum*; *G. barbadense*). Allopolyploid *A. hypogaea* is the only cultivated polyploid *Arachis* species, and one of only two polyploid species in the botanical section *Arachis* (the other being the biologically conspecific *A. monticola* Krapov. & Rigoni). Furthermore, *A. hypogaea* is most likely derived from only one hybridization event and so within-species genetic diversity is narrow (Kochert et al., 1991, Moretzsohn et al., 2013). There is a paucity of large-scale SNP discovery sets in peanut and of those, false-positive rates have been high (Khera et al., 2013, Zhou et al., 2014). Using ddRADseq (double-digest restriction site associated DNA sequence) libraries, Zhou et al. (2014) generated almost one billion paired-end reads to genotype a recombinant inbred line (RIL) population of 166 individuals to identify only 1621 mappable SNPs. More efficient genotyping efforts in peanut must rely on lower cost, more routine, high-throughput genotyping strategies. An SNP array, flexible for use in different *Arachis* mapping populations will be essential to provide researchers worldwide with the power to harness genomics to improve peanut.

Here, we report deployment of the first large-scale SNP genotyping array for *Arachis* to assess allelic diversity between cultivated/wild germplasm and among cultivated sets of germplasm representing global diversity on the one hand and focused breeding programs on the other. Cultivated germplasm with importance in India, Israel, Africa, and the United States was utilized in this study. SNPs from diploid wild species were included for flexibility in genotyping interspecific hybrid populations. The utility of this array was demonstrated by assaying 384 genotypes, including elite US germplasm, the USDA mini core germplasm collection, interspecific hybrids, diploid wild species, and *A. hypogaea* RIL populations. By genetically following the breeding history of US runner market types, which are the most widely grown and economically important in the United States, genomic regions for which breeders have positively selected were unveiled. By sampling these genotypes of direct relevance to breeders, the efficiency of cultivated × cultivated crosses for the development of future populations for gene discovery can be predicted.

## Results

An Affymetrix genotyping array was developed using a set of 58 233 putative SNP markers (Supplemental Table 1 and Pandey et al., 2017). The array was designed to be highly flexible for *Arachis*, with applications for genotyping *A. hypogaea* × *A. hypogaea* populations, interspecific populations, and intraspecific diploid populations. Specifically, the array was designed to include 21 547 and 22 933 markers from *A. hypogaea* identified relative to the *A. duranensis* (A) and *A. ipaensis* (B) genomes, respectively. The set of *A. hypogaea* genotypes used was selected to include important parents of research community RIL populations, segregating for disease resistance, biotic stress tolerance, pod characteristics, and yield, varieties identified as being aflatoxin resistant and drought tolerant, as well as globally important cultivars and parental genotypes important in Israel and India (Supplemental Table 2). The array included 21 validated markers that are being used to select for fatty acid composition and resistance to late leaf spot and rust. In addition, 13 732 markers were included from diploid species, including 3384 polymorphic within *A. duranensis* species, 2389 and 2195 markers polymorphic between A genome species *A. stenosperma* and *A. cardenasii*, respectively, and *A. duranensis*, and 2709 and 2605 markers polymorphic between B-genome-compatible species *A. batizocoi* and *A. magna* Krapov., W.C. Greg. & C.E. Simpson, respectively, and *A. ipaensis* (Supplemental Table 3). More details on this array are provided in Supplemental Results.

### Markers Detecting Tetrasomic Recombination

A critical observation has recently been made that tetraploid peanut, once assumed to always exhibit disomic inheritance as an allotetraploid, harbors regions of the genome that can exhibit tetrasomic inheritance (Leal-Bertioli et al., 2015a, Leal-Bertioli et al., 2015b). This observation was made by the presence of unexpected genotypes of microsatellite and SNP markers. This array could possibly contain markers that could more specifically define these regions of tetrasomic recombination. This is important information for peanut geneticists because these regions are located in the telomeric/gene-rich regions of the chromosomes, and standard genetic mapping will disregard these regions if this phenomenon is not accounted for. To identify tetrasomic markers, the clustering of the 15 897 tetraploid markers was manually inspected using the tetraploid genotypes in the mini core collection and the 64 additional released cultivars and breeding lines. The first criterion used to investigate a marker was excessive heterozygosity, which we defined as more than one third of the genotypes being scored as heterozygous. All of these lines are highly inbred and residual heterozygosity will be very low and not shared by many genotypes. A marker that showed excessive heterozygosity was inspected for unexpected clusters. A “tetrasomic marker” will show a small number of genotypes scored either as “OTV” (off-target variant) or as the opposite homozygous call. The clusters, however, will not be aligned like a disomic marker with many heterozygous individuals. Instead, the two clusters with the majority of individuals will be clustered close together and the third will be much further away. Room for missing clusters representing the additional tetrasomic genotype classes is obvious from the empty space, but the assumption is that no genotypes representing these classes were present in the dataset. In this case, the markers in the physical vicinity were evaluated and checked to determine if the accessions were consistent, indicating a larger region that had undergone tetrasomic recombination.

**Figure 1:**
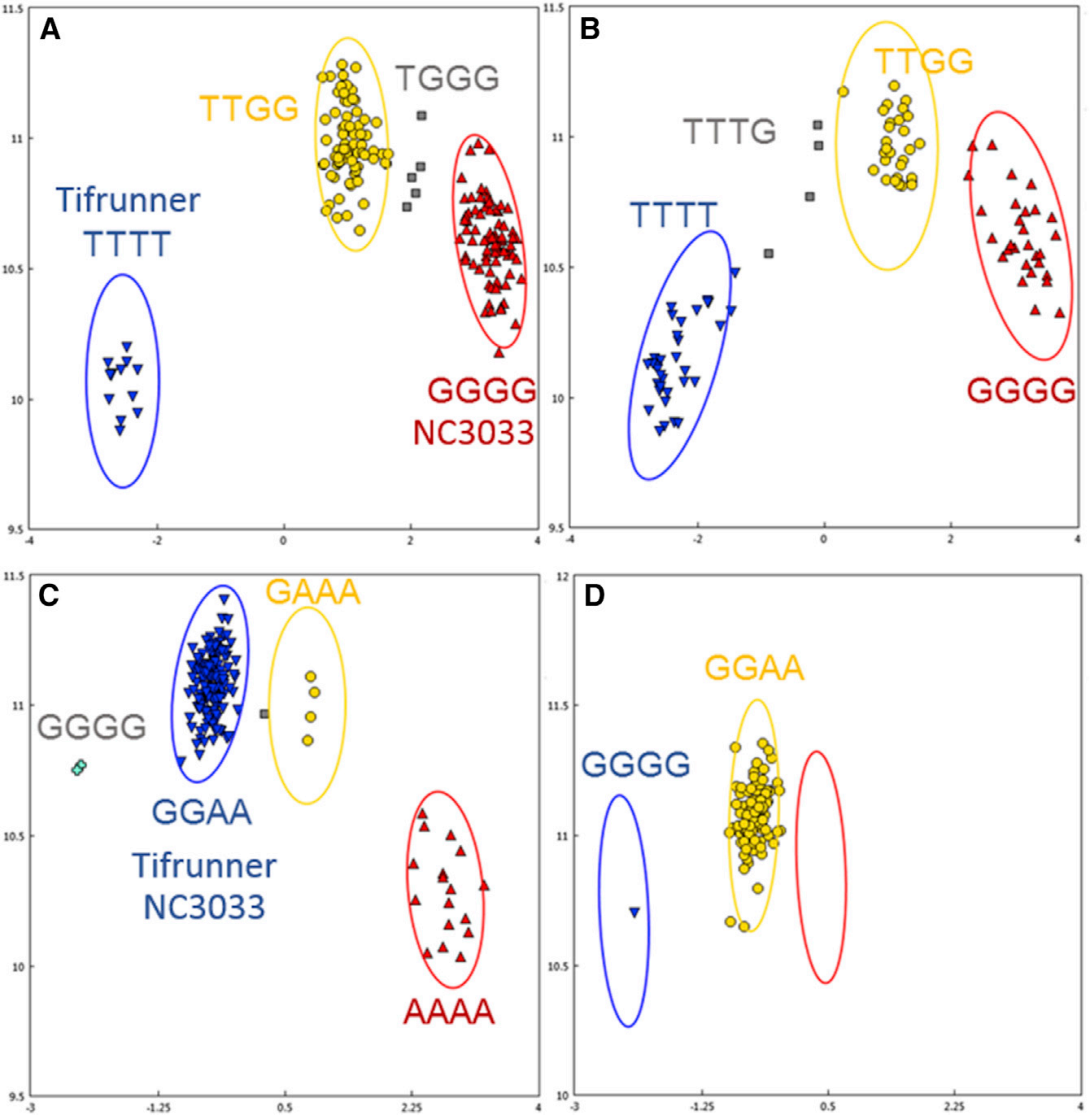
Tetrasomic Markers in Tetraploid Germplasm and Segregating in RIL Populations. **(A and B)** Tetrasomic segregation in tetraploid germplasm where Tifrunner and NC3033 are homozygous quadriplex and nulliplex **(A)** and segregation in the RIL population showing tetrasomic segregation **(B)**. **(C and D)** Tetrasomic segregation in tetraploid germplasm where Tifrunner and NC3033 are monomorphic **(C)** and RIL population showing tetrasomic recombination in one individual **(D)**.

A set of 1193 markers with segregation indicative of tetrasomic recombination were identified (Supplemental Table 4). All the signals in this set represent tetrasomic recombination events, although some detected in Tifguard are probably indicative of alien diploid introgression (Nagy et al., 2010). To investigate these tetrasomic markers further, individuals were sampled from the F_6:8_ RIL population originating from a cross between Tifrunner and NC3033. Tifrunner and NC3033 have large tetrasomic regions on chromosome 4 (Tifrunner) and chromosome 6 (NC3033) (Supplemental Table 4). This information was used to investigate how these regions segregate in a bi-parental population. Figure 1A shows a tetrasomic marker from chromosome A04 when analyzed with the panel of tetraploid genotypes and the same marker within the RIL population in Figure 1B. Tifrunner has the genotype score for T^a^T^a^T^b^T^b^ where NC3033 is scored as G^a^G^a^G^b^G^b^. In the panel of tetraploid genotypes, four of the expected five tetrasomic clusters are present: quadriplex for G, quadriplex for T, monoplex for G, and duplex. In the RIL population, four of the clusters are present, with residual heterozygosity in the form of triplex for T.

Figure 1C and 1D show a representative tetrasomic marker where both parents have the same genotype, A^a^A^a^G^b^G^b^. In this case, in the RIL population, one individual has undergone tetrasomic recombination (Figure 1D). This observation is consistent with similar observations in RIL populations (Leal-Bertioli et al., 2015a, Bertioli et al., 2016), indicating that tetrasomic recombination events in these regions are relatively rare and are only detectable at low rates even after a few generations.

**Figure 2:**
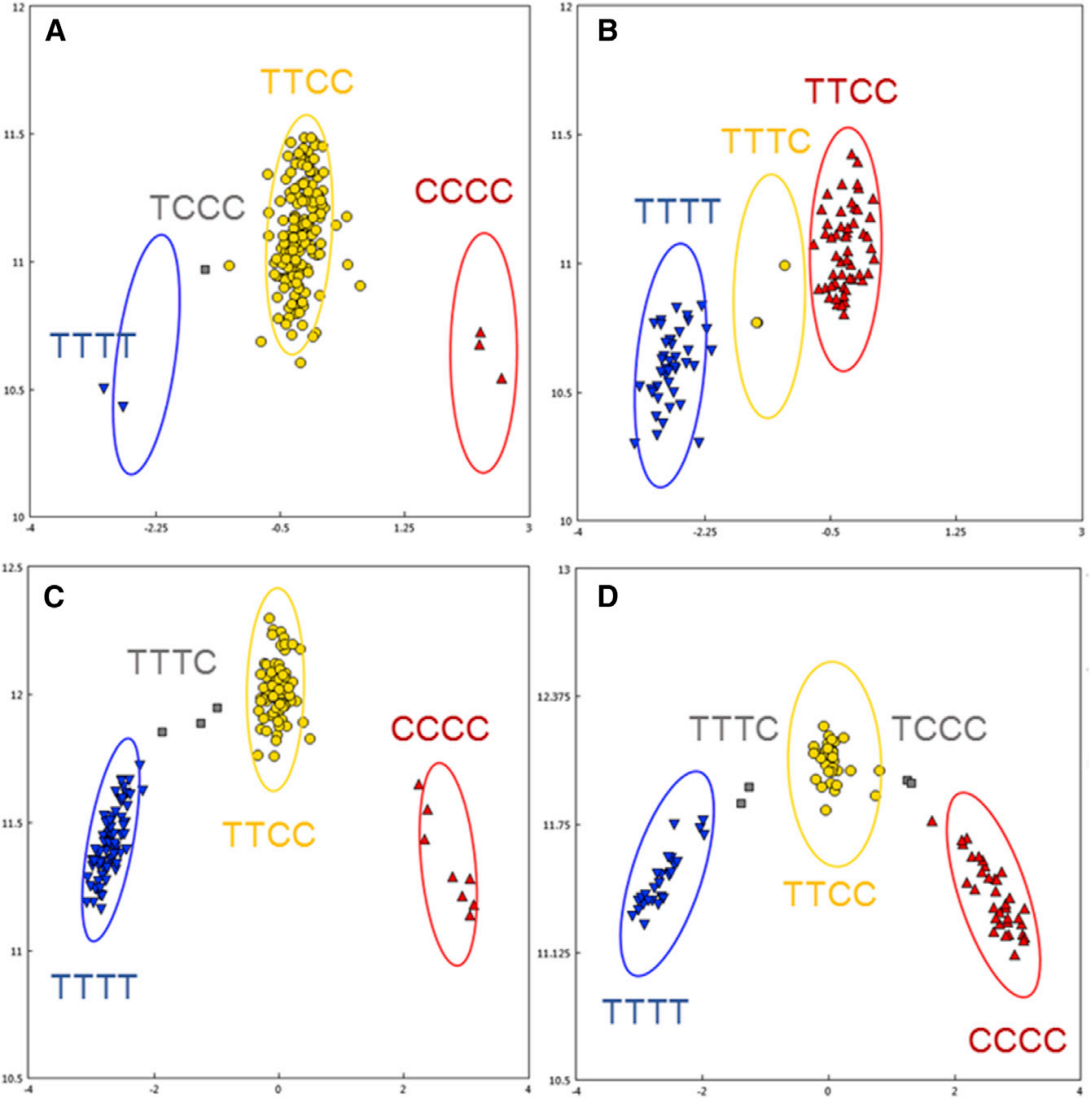
Tetrasomic Markers in Tetraploid Germplasm and Segregating in RIL Populations. **(A and B)** Tetrasomic segregation in tetraploid germplasm where Tifrunner and NC3033 are homozygous quadriplex and duplex **(A)** and segregation in the RIL population showing disomic segregation **(B)**. **(C and D)** Tetrasomic segregation in tetraploid germplasm where Tifrunner and NC3033 are quadriplex and nulliplex **(C)** and the RIL population showing tetrasomic recombination **(D)**.

Further inspection of the 109 tetrasomic markers on chromosomes A04 and A06 segregating in the RIL population originating from the cross between Tifrunner and NC3033 showed that novel allele dosages on chromosome 4 originate from Tifrunner and those on chromosome 6 from NC3033. This provided an opportunity to study the segregation of these markers in a bi-parental population. Parental genotypes were inspected in order to predict the segregation patterns in the population. If the parents were quadriplex and nulliplex for a particular allele, we predicted that we would see excessive heterozygosity in the population, as the heterozygous scores are actually duplex genotypes. If one parent is quadriplex and the other is duplex, segregation should appear as disomic, with very few heterozygous genotype calls. Figure 2A–2D shows two examples. Figure 2A and 2C shows marker behavior with the panel of tetraploid genotypes. In Figure 2A, the parents are quadriplex and duplex. In Figure 2C, the parents are quadriplex and nulliplex. Figure 2B and 2D shows the same markers in the RIL population. As expected, Figure 2B shows a disomic segregation pattern. Figure 2D shows a tetrasomic segregation pattern with all five expected clusters represented.

It is important to distinguish between markers that are detecting tetrasomic recombination in real time and double-dosage markers that are detecting tetrasomic recombination that has originated at some point in the past. Whereas the marker shown in Figure 1C and 1D highlights a marker that has detected tetrasomic recombination within the RIL population, the other examples are markers that are detecting older tetrasomic recombination that are now presented as double-dosage markers. These findings show that this array is capable of resolving these regions of the genome for accurate genotyping of populations.

### Tracking Recombination and Genetic Diversity in US Runner Market-type Cultivars

**Figure 3:**
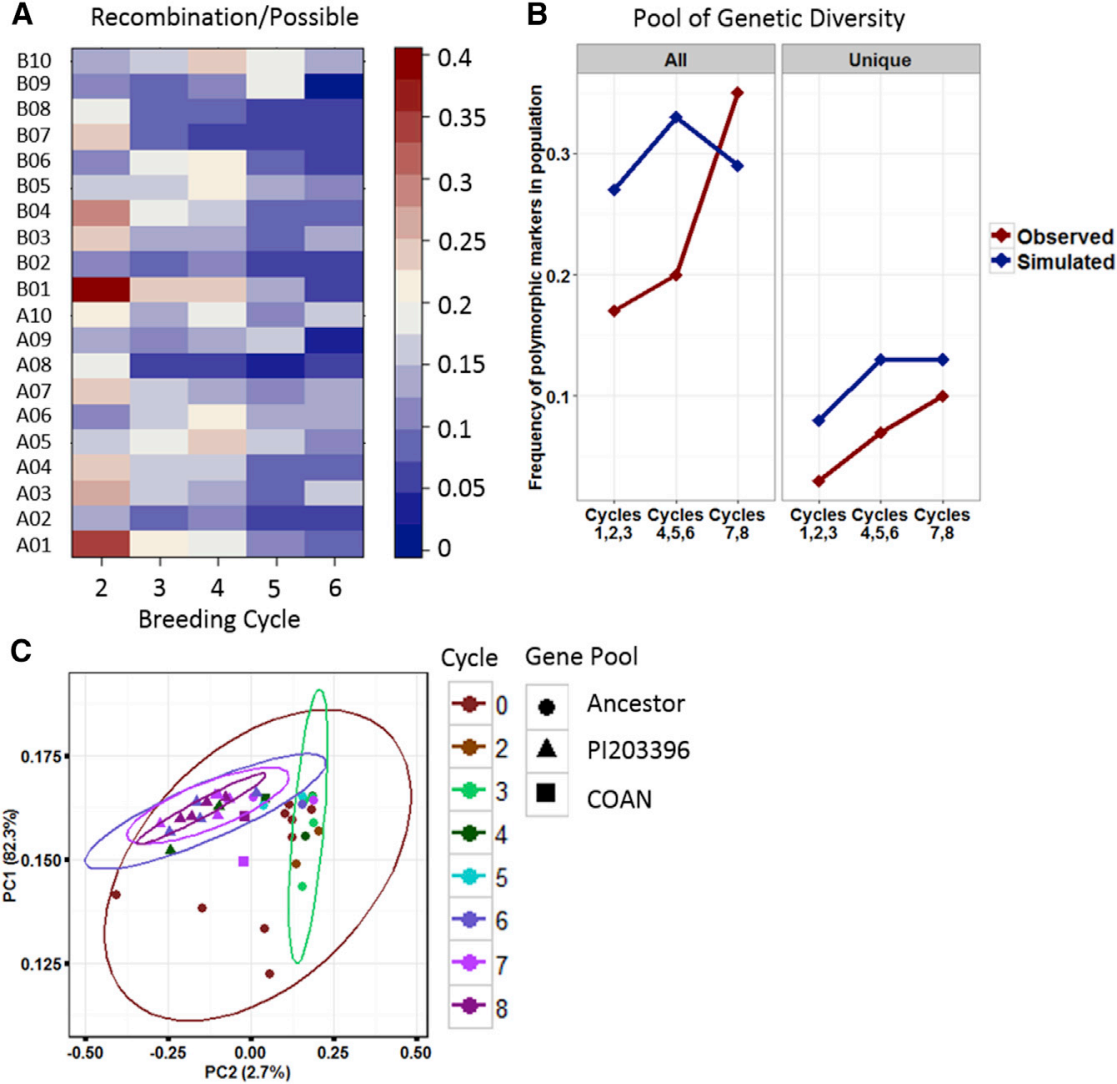
Tracking Changes in Recombination and Genetic Diversity in US Runner Genotypes. **(A)** Recombination events/number of possible events for each chromosome grouped by breeding cycle. **(B)** Frequency of all polymorphic markers (left panel) observed in the populations and 1% of the simulated distribution of simulated polymorphism due to genetic drift. The right panel shows markers unique to only one ancestor. **(C)** First two principal components of genetic diversity between cultivars grouped by breeding cycle and major germplasm introduction.

The sampling allowed tracking of 11 breeding paths ranging from three to five cycles to look at recombination events over the history of breeding. Recombination was defined as a new phase change between adjacent marker polymorphisms that are able to be measured. This is distinguished from recombination and gene conversion that the SNP array cannot measure. The assumption is that the released cultivars make up sampled individuals from a real-world MAGIC (multi-parent advanced generation intercross) population, and one can see how many novel recombination events occurred after six cycles of breeding. Figure 3A shows the average recombination events/opportunities for recombination for each chromosome and each cycle of breeding from cycle two to cycle six. Each cycle represents a cross, followed by cycles of inbreeding and selection. The final released selection is the genotype that was sampled for recombination. This genotype represents the theoretical best possible combination of marker polymorphisms from this cross given the sample size, selection criteria, and phenotyping methods. There was a drop off in recombination after the fourth cycle, from 15% of marker pairs showing recombination to 11% and 9% in the fifth and sixth cycles, respectively. The distance between markers with sampled recombination at each cycle was investigated to indicate the power of breaking blocks of linkage disequilibrium. Supplemental Figure 1 shows the distance between recombination events from one to five crosses. The minimum distance occurs after one and two crosses and rises significantly after each cross from three to five. These data indicate that in peanut, a MAGIC population may be maximized more by population size than number of crosses if crossing elite cultivars with each other. The tendency of rare/unique (only occurring between two markers after one cross) recombination events to occur at each cycle of crossing was investigated (Supplemental Figure 1). The distance between rare/unique recombination events is similar to all recombination events. The lowest distance between markers that show a phase change from the parental haplotype occurs after one and two crosses and increases after the third cross. For rare/unique events, there is no change after three crosses as the distributions of distance between markers does not change. The average number of rare/unique recombination events after each cross is consistent with these results, as the maximum number occurred after two crosses and decreased steadily after each subsequent cross. The average number of rare/unique recombination events occurring after five crosses decreased to only 83 (Supplemental Figure 2).

Each cycle of breeding was tracked for the percentage of fixed SNPs compared with the percentage of marker polymorphisms that could recombine in unique haplotypes. Of the 15 897 possible tetraploid markers on the array, 12 477 (78%) were fixed among the four main ancestors: Basse, Spanish 18-38, Dixie Giant, and Small White Spanish. The other ancestors donating significantly to runner germplasm, PI203396, Jenkins Jumbo, and Virginia Bunch 67, contributed an additional 1022, 230, and 142 new marker polymorphisms, respectively. These introductions came into the runner germplasm at cycles three (Jenkins Jumbo), four (PI203396), and five (Virginia Bunch 67). Cultivars released in cycles one, two, and three were fixed for 83% of markers. From new introductions, the amount of fixation decreased in cycles four to eight (80% at cycles four, five, six and 65% at cycles seven and eight). If looked at as pairwise shared identity between cultivars, many of the retained polymorphic markers have low minor allele frequencies. Cultivars released in cycles one, two, and three share on average 92% of alleles, up from 84% in the ancestors. The shared identity increases in cycles four, five, and six to 93% and then decreases slightly to 91% in cycles seven and eight. So, although there is increased polymorphism among the released cultivars in terms of total alleles, there has been an overall reduction in pairwise diversity. It is possible that the available genetic diversity was lost due to genetic drift as a consequence of small population sizes and inbreeding. To investigate whether the allele fixation observed in the historical breeding cycles can be attributed to more than what would be expected due to genetic drift, a simulation was carried out that recreated the historical pedigree with no selection of progeny as released cultivars and random mating of cycle progeny where no ancestor was used as a parent (cycles six, seven, and eight). Figure 3B shows the simulated 1% of allele fixation at each cycle along with the observed values of all polymorphic markers (Figure 3B; left panel) and markers unique to only one ancestor (Figure 3B; right panel). For all markers, an extreme bottleneck in cycles one, two, and three leads to polymorphism below what can be attributed to genetic drift. Despite the inclusion of new alleles in cycles four, five, and six, the polymorphism still remains lower than expected. In cycles seven and eight, the polymorphism increases above the expected level, indicating that breeders have begun increasing the diversity in recently released cultivars. The levels of polymorphic markers that are unique to a specific ancestor have remained below that which can be attributed to drift (Figure 3B; right panel). An investigation into the allele frequency distribution of these ancestor-specific alleles in the mini core collection, which includes the estimated genetic diversity for *A. hypogaea*, indicates that these alleles are in general rare, as over 50% of them have an allele frequency below 0.05 (Supplemental Figure 3). Despite this, there is a relationship between mini core frequency and frequency in released cultivars, where the average change in frequency among ancestor-specific SNPs is essentially zero (0.02).

Genetic diversity in the runner germplasm can be further investigated using principal components. Figure 3C shows a principal components analysis of all of the cultivars assayed grouped first by the breeding cycle from which they were released and then by major ancestor. We chose as ancestors the main four as a group, PI203396 which still accounts for 29% of marker polymorphisms in progeny two crosses removed from it, and COAN which introduced *A. cardenasii*-derived alien alleles into the germplasm (Simpson and Starr, 2001). The first principal component accounts for 82% of the variance and the second only 2%. Breeding cycles zero, two, and three maintain the variance accounted for by PC1. Cycles five, six, and seven decrease that diversity as the cultivars released from those later cycles become delineated more by PC2, then PC1. These three analyses combined show that the genetic diversity within cultivated peanut has been decreased to a level where few new effective recombination events occur and the level of fixed marker polymorphisms has increased to an estimated 98%.

### Signatures of Selection from US Runner Market-type Breeding Programs

Before the 1940s, most of the peanuts grown in the United States were Spanish types (*A. hypogaea* ssp. *fastigiata* var. *vulgaris*). The first runner-type peanut released was Early Runner in 1952 by W.A. Carver (Isleib et al., 2001). Florispan, released in 1953, became the ancestor of many of the current peanut cultivars through its progeny F435, Florunner, and Florigiant (Isleib et al., 2001). Breeders have made significant improvements in yield, grade, and seed size over the history of US breeding programs. Knowledge of the pedigree of the released cultivars allows identification of regions of the genome that have undergone significant selection since intensive breeding began in the 1930s.

There were a total of 5537 polymorphic markers on the array among the 64 cultivars and breeding lines we assayed. Directed selection of a particular allele was first examined when two parents were crossed, and a selection was made from that population for release as a new cultivar. For each marker, selection was assessed using a binomial exact test for distorted segregation given an expectation of a 1:1 ratio given random selection. To control for the effect of genetic drift from inbreeding and small effective population sizes inherent in the breeding process, a simulation was carried out assuming random selection. This simulation attempts to account for the loss of alleles due to drift and rapid fixation in earlier cycles. Three different F_2_ population sizes (200, 300, 400) were used with three different selection intensities (0.1, 0.2, 0.3), and each combination was simulated 553 700 times (100 simulations per marker) and the 99th percentile of the distribution for all number of tests from 0 to 45 is shown where a test is a polymorphic parent between the two sample parents (Supplemental Table 5).

**Figure 4:**
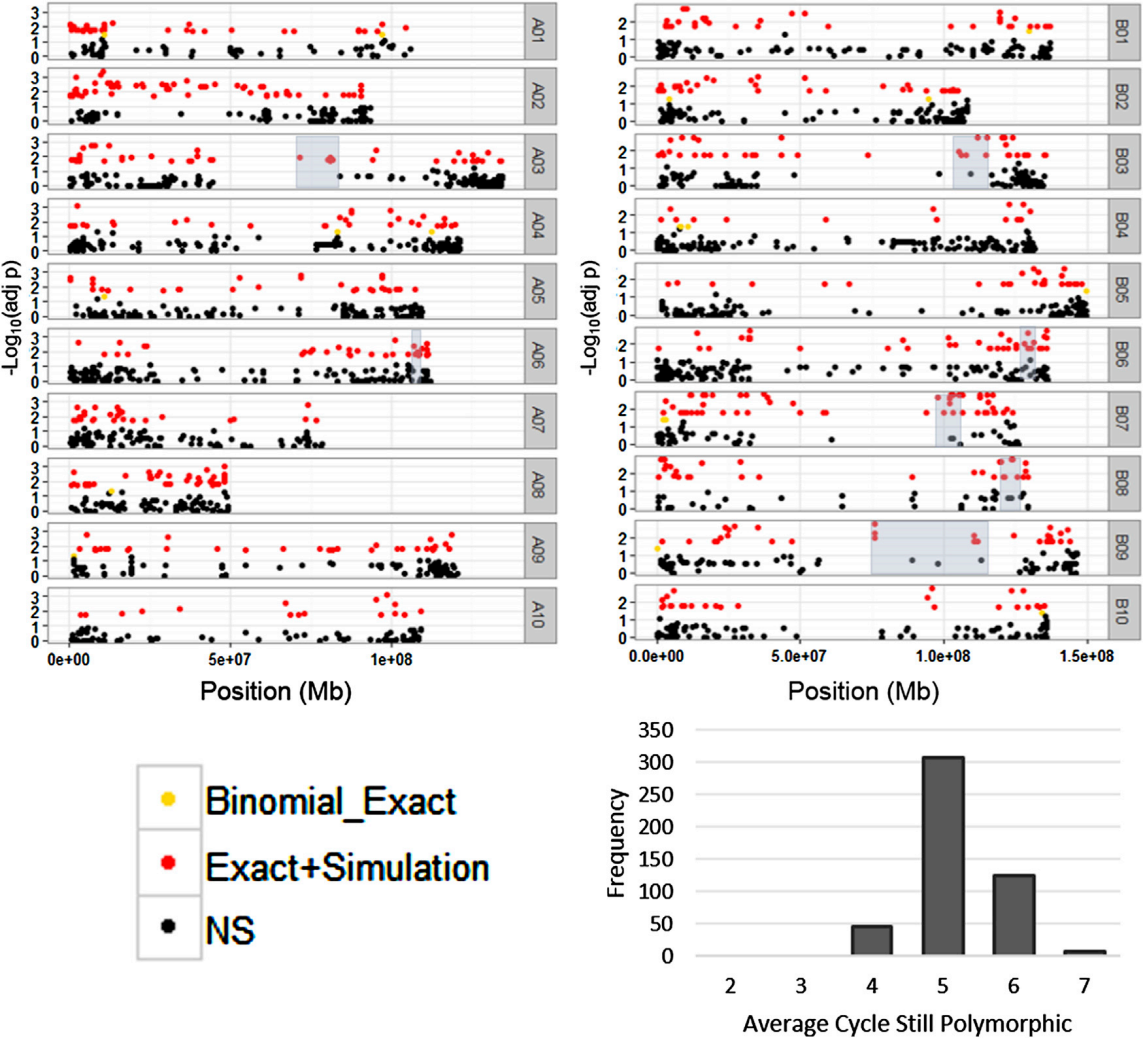
Analysis of Breeding Trios Uncovers Signatures of Selection. Log transformed *p* value of the binomial exact test of directed selection versus no selection by physical position of *A. duranensis* (A genome, left panel) and *A. ipaensis* (B genome, right panel) pseudomolecules.

There were 775 markers that were significant by both tests (14%) but, taking into account probable linkage disequilibrium, there were only 267 unique genomic regions (4.82%) (Figure 4; Supplemental Table 6). The frequency of the average cycle where these 485 markers were still polymorphic shows a peak between cycles four and five (Figure 4; Supplemental Table 6). To investigate these loci further, pairwise haplotype sharing (PHS) was calculated and normalized by the ancestor genotypes (Supplemental Table 6). There were 37 significant loci with outlier PHS values within the block that was greater than the 99th percentile of the distribution of all PHS values (>8.11 for cycles 4, 5, and 6 and >9.68 for cycles 7 and 8). An enrichment test was conducted to test the distribution of GO terms within significant loci against the distribution of GO terms genome wide. Seven genomic regions were selected that included more than five significant markers in succession, with not more than one non-significant marker in between two significant markers, and at least one marker with an outlier PHS value (Supplemental Table 7; Figure 4, shaded boxes). Of those seven, five did not contain enough genes to test. Two regions containing the most consecutive significant markers also show enrichment of interesting GO terms. A region spanning 119 671 975 to 127 778 744 on chromosome B08 shows enrichment for recognition to pollen, specifically five genes containing the S-locus glycoprotein domain. Finally, a region spanning 75 923 207 to 124 434 201 on chromosome B09 shows enrichment for a defense response, with 13 Miraculin-like protein (MLP-like) genes and a small cluster of seven TIR-NBS-LRR class resistance genes.

Maturity is an important trait in all crop plants, including peanut, and breeders may have preferentially selected for this trait to meet the needs of growers. Four well-studied maturity genes from soybean were examined, *E1* (Xia et al., 2012), *E2* (Watanabe et al., 2011), *E3*, and *E4*. Using BLASTx, putative peanut orthologs were identified using the *A. duranensis* and *A. ipaensis* gene models (Bertioli et al., 2016) (Table 1). With the exception of *E3*, at least one homeolog of all the maturity genes was located in genomic regions that showed directed selection (Table 1). A recent study in soybean showed similar evidence of selection in released lines around these maturity genes (Vaughn and Li, 2016). As would be expected, the regions surrounding *FAD2A* (A09) and *FAD2B* (B09), genes that have mutations that when combined confer a high oleic acid ratio (Jung et al., 2000), contain significant SNPs, although on chromosome A09 the causative SNP is only significant by the binomial exact test, because it became fixed in early cycles and there were not enough tests (12) to reach the power of the simulated thresholds. The region of *FAD2B*, however, remained polymorphic on average until between cycles five and six, and the nearest marker shows an outlier PHS value of 33.91 (Table 1). This is intuitive, because the underlying mutation was introduced through F435 in cycle four and has been selected for recently using marker-assisted selection (Supplemental Figure 4).

### New Germplasm Introductions Retain Specific-Allele Frequency in Modern Cultivars

Specific alleles from important ancestors were tracked through seven cycles of breeding and retention was noted at each cycle. A set of SNPs was tracked specific to the subsp. *fastigiata* ancestor Small White Spanish and Spanish 18-38. Cultivars were assigned cycles based on Isleib et al. (2001) as the number of cycles of crossing and selection of a cultivar was removed from its founding ancestors. The seventh cycle ending with the cultivars Georgia Greener, Georgia-06G, Georgia-07W, Georgia-01R, and Tifguard. Georgia-06G, released in 2006, is still the dominant cultivar grown in the southeast. The US runner germplasm can be divided into two main groups: pre-Florunner and post Tomato Spotted Wilt Virus (TSWV). Florunner was a selection from a backcross of Florispan to one of its parents, Early Runner, and so all alleles from Florunner came from the four main founder ancestors Basse, Spanish 18-38, Dixie Giant, and Small White Spanish. Florunner dominated peanut production for two decades from 1972 to 1993. From 1976 to 1984, Florunner occupied 95% of the acreage in Georgia, Florida, and Alabama (Isleib et al., 2001). Specific alleles from Small White Spanish were highly maintained in Florunner, with 75% unique alleles present in the cultivar (Supplemental Table 8). Other ancestors were selected against heavily, with only 5%, 2%, and 16% of unique alleles from Basse, Spanish 18-38, and Dixie Giant, respectively, maintained in Florunner (Supplemental Table 2). Florunner was routinely used as a parent for new cultivars and its genetic signature was spread across breeding programs. The decline of Florunner was due to its susceptibility to TSWV, which became a major problem in the 1990s. To combat TSWV, breeders introduced resistance from PI203396, a collection from Brazil in 1952. This introduction into the germplasm had a rapid and profound effect on the genetic makeup of US runner cultivars. Introduced in cycle four, the cultivars Southern Runner and Tifrunner maintained 40% of PI203396-specific alleles while the alleles from Small White Spanish were selected against, maintaining only 13%. These alleles from Small White Spanish, maintained through three cycles of breeding and grown in the field for more than six decades, were reduced from 75% to 13% in one breeding cycle. After TSWV became a problem, there was a shift to Georgia Green, which included resistance from PI203396 and has early maturity compared with Southern Runner. Interestingly, Georgia Green has the smallest proportion of PI203396-specific alleles (13%) while C99R maintains 34%. Georgia-06G, the dominant variety in the southeast presently, has as its parents Georgia Green and C99R and maintains 23% of PI203396 alleles. Overall, cultivars released from cycles seven and eight maintain an average of 29% and 28%, respectively, of PI203396-specific alleles. This is by the far the most impact of any ancestor on modern runner cultivars grown in the southeast.

### Haplotype Frequency Analysis Shows Selection of Novel Haplotypes in Recent Cultivars

As described above, genetic diversity in terms of SNP markers has decreased more than can be attributed to genetic drift during modern runner-type peanut breeding. The 111 mini core genotypes that estimate the breadth of diversity in *A. hypogaea* in the USDA collection were used to identify all possible haplotypes and rank them according to frequency. The haplotype from PI203396 (due to its importance to modern peanut) and the top eight haplotypes ranked by frequency were assessed for frequency in the eight ancestors, cultivars released in cycles four, five, and six, and cultivars released in cycles seven and eight. In addition, the haplotype diversity (π) was assessed as the pairwise average nucleotide diversity across the 20 marker haplotypes and normalized to the diversity in the mini core collection by taking the log of π_population_/π_mini core_, which shows a decrease in diversity as a negative value.

**Figure 5:**
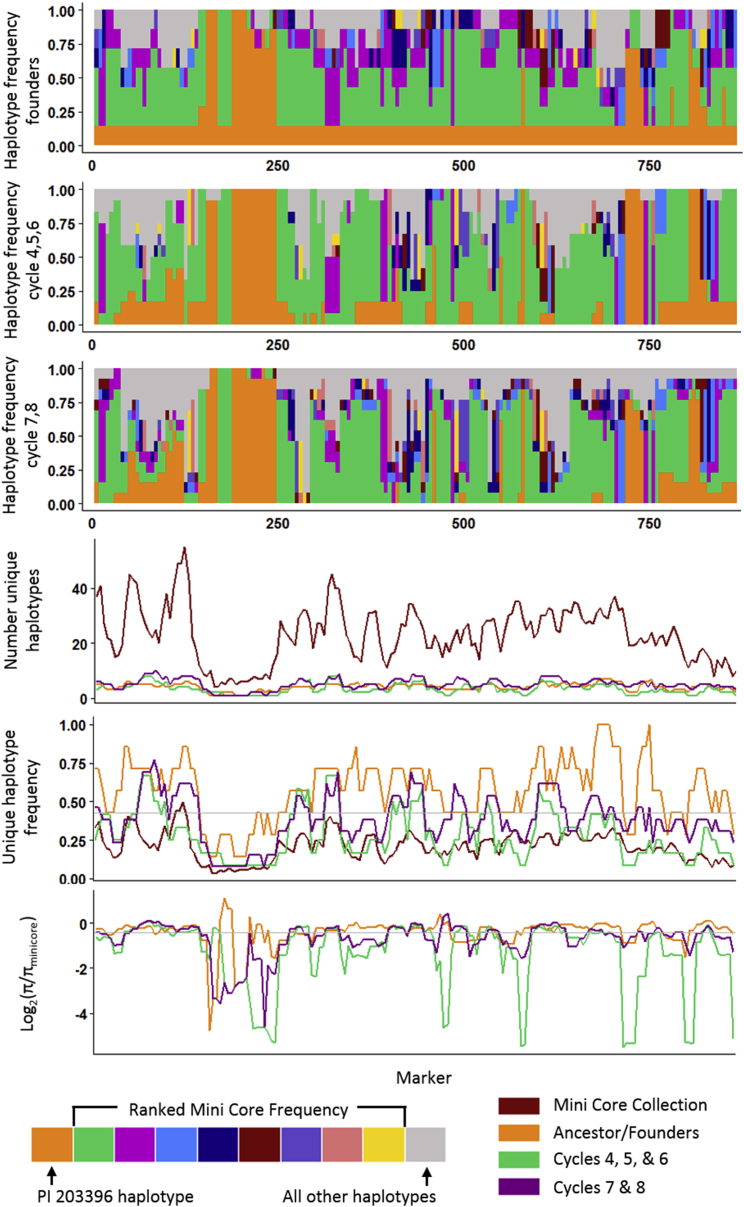
Haplotype Frequency and Diversity on Chromosome B09. Top three panels: Haplotype frequency was determined in the USDA mini core collection in 20 marker sliding windows moving five marker intervals. The top eight haplotypes in terms of frequency along with the haplotype from PI203396 were then assessed for frequency in the eight main ancestors (top), cultivars released in cycles four, five, and six (top middle), and cultivars released in cycles seven and eight (top bottom). Line graphs below show the number of unique haplotypes in the four populations, the ratio of unique haplotypes to population size, and genetic diversity normalized to the estimated *A. hypogaea* haplotype diversity from the mini core collection as log_2_(π population/π mini core).

The haplotype frequencies paint an encouraging picture compared with SNP marker diversity (Figure 5 chromosome B09; all other chromosomes in Supplemental Figure 5). The cultivars released in cycles four, five, and six show low haplotype diversity shown by frequency and peaks of decreased diversity. The expected further decrease in cycles seven and eight, however, does not take place. There are a few regions where haplotype diversity is reduced compared with the ancestors and is further reduced within the two populations, but in most cases the diversity is reduced in cycle four, five, and six cultivars, but then is increased again in cycle seven and eight cultivars (Figure 5; Supplemental Figure 5). This observation suggests that although SNP diversity has decreased, breeders have selected for new haplotypes in modern cultivars and maintained haplotype diversity.

## Discussion

Discovery of high-quality SNPs while minimizing false positives has been challenging in peanut due to genome complexity. Large-scale validation of SNPs identified from next-generation sequence data has seen error rates between 86% and 93.8% (Khera et al., 2013, Zhou et al., 2014, Peng et al., 2016, Zhao et al., 2016). A pipeline, SWEEP, that uses putative homologous polymorphisms as anchors to identify true SNPs, was developed (Clevenger and Ozias-Akins, 2015) and was used for SNP identification from re-sequencing data of 20 *A. hypogaea* accessions. The initial validation of SWEEP-filtered SNPs was done using RNA-sequencing data, which as a strategy for genome reduction is less sensitive to false positives stemming from repetitive DNA in non-genic regions. Coding sequence, however, is highly conserved and contains less polymorphism among genotypes. For large-scale SNP discovery in peanut, the genomic sequence needed to be assayed. The accessions used for SNP discovery were on this array, allowing for unprecedented SNP validation in peanut. Although 33% of tetraploid SNPs were validated on the array, this was more than double the accuracy previously achieved. However, a vast improvement in methodology is still needed to apply next-generation sequencing to sequencing-based genotyping methods, and validated SNPs will provide a training set to improve our methods. We have 14 233 SNPs identified with re-sequencing data and validated on the array, and 28 425 false-positive SNPs identified in the same dataset. This allows deciphering the differences between true SNPs and false positives in real peanut data on a large scale and will provide the key insights needed to train models to achieve higher accuracy.

### A Truly Flexible Genotyping Tool

The goal in designing this array, a first-generation SNP genotyping array for *Arachis* species, was to address the needs of all researchers working in *Arachis* genetics, breeding, and improvement. The future of peanut breeding is likely to rely heavily on the inclusion of disease and stress tolerance alleles from wild species, and the genotyping of interspecific hybrid populations will be crucial to map, identify, and transfer these desirable alleles (Stalker, 1984, Lyerly et al., 2002, Foncéka et al., 2009; Leal-Bertioli et al., 2015b). Despite the decreasing genetic diversity in the elite germplasm of tetraploid peanut, there is a wealth of tetraploid genetic resources in germplasm collections that has been untapped, although runner types are currently underrepresented. Fine work from peanut geneticists has shown that there are beneficial alleles in the tetraploid germplasm collection (Anderson et al., 1993, 1996; Holbrook and Isleib, 2001, Damicone et al., 2010), so mapping these yet to be discovered desirable alleles will necessitate good markers segregating in tetraploid germplasm.

There are 15 897 polymorphic tetraploid markers on the array giving more markers per cross than peanut researchers previously have used for genetic mapping studies (Qin et al., 2012; Sujay et al., 2012; Zhou et al., 2014; Huang et al., 2015) and in consensus SSR maps (Gautami et al., 2012; Shirasawa et al., 2013). Zhou et al. (2014) constructed an SNP linkage map comprising 1621 markers, but this number of mappable markers in a population of 166 individuals required the generation of close to 1 billion paired-end reads. As a comparison, the Tifrunner × NC3033 RIL population genotyped here generated 2226 segregating markers. Producing the array data does not require construction of sequencing libraries or processing of next-generation sequence data for all individuals and most importantly does not necessitate analysis by highly skilled bioinformaticists.

Perhaps more intriguing is the application of the array for genotyping interspecific populations. Polymorphic markers were scored between three A- by B-genome-compatible induced allotetraploids (*A. ipaensis* × *A. duranensis*; *A. batizocoi* × *A. stenosperma*; *A. gregoryii* × *A. stenosperma*) and three elite cultivars that have been and could potentially be used as recurrent parents in interspecific populations (Florunner; Tifguard; Georgia-06G). The number of polymorphic markers on the array will be very useful for genotyping these populations with a range of 9924 and 29 748 polymorphic markers between these cultivars and the interspecific hybrids. This is an increase in markers for mapping by 268%–800% from 3693 SSR markers in a consensus map integrated from 13 published maps (Shirasawa et al., 2013). The future of peanut cultivars will likely rely more heavily on introgressed wild alleles for increased disease and stress tolerance, and this array will efficiently facilitate the identification and introgression of these alleles.

### Mapping Regions of Tetrasomic Recombination

Leal-Bertioli et al. (2015a) described and provided evidence for their observation that regions of the *A. hypogaea* genome have and are exhibiting tetrasomic recombination. These specific regions have been largely ignored in peanut genetic maps as markers showing segregation distortion. With next-generation sequencing, these regions can be defined, but at high cost per genotype. We have identified and annotated 1193 markers on the array that are in regions that have undergone tetrasomic recombination and can detect all genotypic classes that arise at these loci. It is unclear what effect these regions have phenotypically. One event, on chromosome 4, is prevalent in subspecies *fastigiata* (valencia type). The same tetrasomic alleles are present in PI 203396, a subspecies *hypogaea* accession collected in Brazil. PI 203396 was an important parent for TSWV resistance in runner germplasm and is an ancestor of many current cultivars (Isleib et al., 2001). The tetrasomic region on chromosome 4, however, was only selected for one time, in the cultivar Tifrunner, and so it is unclear if there is a benefit of this tetrasomic event or not. Perhaps of more importance, Tifrunner was selected to be sequenced as the reference *A. hypogaea* genome. The tetrasomic markers on the array were able to physically map the tetrasomic region in Tifrunner to within 4 kb on one side and 3 kb on the other. The entire region spans approximately 610 kb. This is important information for the assembly of the reference genome sequence. More research on mapping the tetrasomic regions that exist in *A. hypogaea* germplasm, and investigations into how these regions manifest themselves phenotypically will greatly benefit from the fast and efficient genotyping this array affords.

### Signatures of Selection Offer Key Insights into Genetic Diversity

Analysis of runner cultivars released in the almost nine decades of peanut breeding in the United States allowed us to unlock a compelling story of signatures of selection and an increasingly limited pool of genetic variation. Peanut breeding in the southeastern United States can be broken into two eras: pre-TSWV and post-TSWV. TSWV was first reported in Brazil in 1941 (Culbreath and Srinivasan, 2011) and moved its way to Texas in 1971 (Halliwell and Philley, 1974). The virus moved east through Mississippi and Alabama, and by 1990 the first losses attributed to TSWV were reported in Georgia. Losses due to TSWV reached a peak in 1997, contributing to a loss of approximately 40 million dollars in crop value (Bertrand, 1998). The dominant cultivar at the time was Florunner, which is highly susceptible to TSWV (Culbreath et al., 2000, Culbreath and Srinivasan, 2011). Georgia Green, moderately resistant to TSWV, was released in 1995 (Branch, 1996), and began to take over acreage from Florunner (Culbreath and Srinivasan, 2011). This can be seen in the production of certified seed. In 1997, Florunner certified seed occupied 200 acres of certified seed production (0.1%). Georgia Green was already occupying 47 163 acres of certified seed production (43%). In the ensuing years, the certified seed acreage devoted to Georgia Green rose to 74% in 1998 and 71% in 2001. Certified seed production of Florunner disappeared and production of cultivars with PI 203396 in their pedigree occupied 82% of certified seed acreage (georgiacrop.com). The change in cultivars had an immediate, dramatic effect on losses due to TSWV, dropping from more than 40 million to less than 5 million in only 3 years (Culbreath and Srinivasan, 2011). The TSWV resistance was brought in from a USDA germplasm accession, PI 203396, which was collected in Brazil in 1952. It was a parent of Southern Runner, which was a selection that showed superior leaf spot resistance, and was never tested for TSWV resistance (Gorbet et al., 1987). Southern Runner was used as a parent for Georgia Green because of its resistance to leaf spot, and as an amazing twist of fate, inherited the TSWV resistance from PI203396 and Southern Runner. This combination of chance and breeder skill saved the peanut industry in the southeast and drastically altered the genetic makeup of runner cultivars. We traced ancestor alleles through the breeding cycles of runner cultivars. At cycle three, Small White Spanish was the most represented ancestor with over 75% of its specific alleles retained in the cultivar Florunner, compared with 3%–16% retained from the other three ancestors. At cycle four, when the TSWV epidemic occurred, alleles from PI203396 took over, and 24%–50% of PI203396-specific alleles were retained in released cultivars through four additional cycles of breeding. Alleles from Small White Spanish, on the other hand, were reduced from 75% to 10% in one cycle. This story has profound implications on the future of peanut breeding for not only the United States, but worldwide. An introduction from a single germplasm accession not only stabilized an industry in the face of a major disease epidemic, but its alleles have persisted in the releases in four successive cycles from 24% to 30% in cycle eight. Since the inclusion of PI 203396 in 1986, there has not been another major introduction of diversity in the form of an *A. hypogaea* accession in southeastern US runner-type breeding programs. When the next epidemic occurs, will the peanut industry have the genetics in the pipeline to combat it? The story of PI203396 shows that bringing in beneficial alleles to elite germplasm can be fast and efficient.

The extreme bottleneck that released runner cultivars went through makes scans for selection difficult to interpret. Cultivars released in cycles four and five are essentially half sibs, sharing Florunner as a parent. Florunner itself is the product of just two unique crosses. We used two different methods to identify signatures of selection in cultivated runner-type peanut: a pedigree-based method adjusted for drift and PHS. The loci identified as showing a signal from both methods are intriguing as regions that have been selected for inbreeding programs more than can be attributed to drift and show an extreme outlier PHS score. One region, containing a cluster of defense response genes, is of note because the haplotypes in that region are shared with PI203396, which donated a resistance package to elite germplasm that stabilized the industry.

Milla-Lewis et al. (2010) published a study of the genetic diversity in US runner cultivars using 34 SSR markers. They concluded that “runner-type peanut breeders have been successful at increasing levels of diversity among cultivars released in the last three decades of modern plant breeding.” This conclusion was based on a limited number of SSR markers, and reasonable considering those data and, from one perspective, is true. Using SNP markers, however, the picture becomes more complex. From the perspective of the number of polymorphic markers among cultivars, this statement is true, although through cycle six the percentage of fixed markers is greater than would be expected due to genetic drift alone (Figure 3B); the cultivars released in cycles seven and eight have decreased the percentage of fixed markers to within the expected distribution. The original level of allele fixation can be attributed to the dominance of Florunner as a parent, a cultivar with an already narrow genetic base. The new decrease in allelic fixation can be attributed to only a few germplasm introductions and the selection of novel haplotypes not present in high frequency in earlier cycles.

From a different perspective, the inclusion of unique alleles, defined by alleles introduced by a germplasm introduction not present in the available pool before introduction, has decreased below expectations due to genetic drift (Figure 3B). This has implications for offsetting disease epidemics and dealing with biotic stresses. The polymorphism present among cultivars is just a reshuffling of the alleles already present. While selecting for new haplotypes with available diversity can lead to gains in yield, the exclusion of rare alleles makes it more difficult to deploy resistant/tolerant cultivars in response to new biotic and abiotic stresses. So from this perspective, the genetic diversity remains low. However, the inclusion of new alleles in recent cycles from germplasm collections provides a framework for future germplasm enhancement.

Here, we report deployment of the first large-scale SNP genotyping array for *Arachis* species. The array contains 47 116 high-quality polymorphic markers within tetraploid genotypes and interspecific hybrids, and 15 897 high-quality polymorphic markers within *A. hypogaea* species. Using diploid *Arachis* species, the array contains 54 564 high-quality polymorphic markers. In addition, the array contains 1193 markers that can be used to map genomic regions undergoing tetrasomic recombination. This array was utilized to survey genetic diversity in US runner cultivars, and it was found that genetic diversity decreased significantly. The pedigree of runner cultivars was used to discover genomic regions that have undergone preferential selection by breeders in the past 80 years of US breeding. The Axiom_Arachis array is truly a flexible genotyping tool and, along with the newly released genomes of *A. duranensis* and *A. ipaensis* (Bertioli et al., 2016; peanutbase.org), will usher in a new era of productivity for peanut research and improvement that will affect global food security for years to come.

## Methods

### *Arachis hypogaea* Re-sequencing Data

A group of 20 cultivated genotypes was selected for whole-genome re-sequencing, including 10 parents of RIL populations (Holbrook et al., 2013) and 10 genotypes with different traits of interest for breeding purposes. The sequencing libraries were constructed using the Illumina TruSeq PCR-free kit starting with 2 μg of total DNA isolated from single plants using a QIAGEN DNAeasy Plant mini kit and sheared using Covaris to obtain 550 bp insert size. The libraries were quantified using an Agilent DNA 7500 kit on the Agilent 2100 Bioanalyzer. Paired-end 150 sequences were generated on an Illumina HiSeq 2500 V4 using eight lanes for a complete flow cell. The 20 samples were pooled in groups of 10, and each pool was sequenced in four lanes in order for the yield to be more homogeneous. The sequencing data from Tifrunner were obtained from the Peanut Genome Consortium, providing ∼8.4× coverage from 500 bp insert size, similar to the coverage obtained for the samples in the sequencing with an average of 10×.

The raw sequences were filtered and trimmed using Cutadapt v1.2.1. for adaptor trimming and TrimGalore v0.3.7. for quality trimming. Approximately 88% of high-quality reads were mapped over the two diploid genomes (A and B genomes represented by *A. duranensis* and *A. ipaensis*; Bertioli et al., 2016) with Bowtie2 using default parameters for sensitive local alignment reporting best alignment and zero mismatch in the 20 bp seed. Consequently, very similar overall alignment rates were obtained for both genomes, being 96.7% on average over the *A. duranensis* genome and 96.9% over the *A. ipaensis* genome. The SNP calling between all the genotypes was based on the reference genomes, and filtering of homologous SNPs was carried out following the SWEEP Prime version program (Clevenger and Ozias-Akins, 2015), which uses Samtools v0.1.9 and Bcftools v0.1.9, with default parameters and ultimate option and minimum depth of 5×. Supplemental Table 2 shows the number of reads, number of cleaned reads, percent alignment to the diploid genomes, trait of interest for each genotype, and NCBI accession numbers where the raw sequence has been deposited. See Supplemental Methods for further SNP filtering information.

### Array Final Design

113,787 tetraploid SNPs from whole-genome re-sequencing, 25 000 diploid SNPs, 5025 SNPs from important Indian cultivars J11, JL24, and ICGV91114 (RNA-seq data), and 24 validated trait-linked SNPs were submitted for design of the Affymetrix SNP array, called Axiom_Arachis array (Pandey et al., 2017). All A/T and C/G SNPs were disregarded because they would require more than two probes on the array. Of the 66 924 markers recommended for tiling on both strands, 58 233 final markers distributed as much as possible across the diploid genomes were chosen. Supplemental Figure 6 shows genome-wide SNP density and predicted polymorphism for the final design.

### Samples Genotyped on the Array

Three hundred and eighty-four samples were genotyped on the Axiom_Arachis array, including 109 accessions of the USDA mini core collection, 64 tetraploid genotypes representing the history of US runner market-type breeding, the 20 parents re-sequenced for SNP selection plus Tifrunner, and important lines harboring traits of interest related to aflatoxin contamination, drought tolerance, and disease resistance, and selections from two F_6:8_ RIL populations with the parents Tifrunner × NC3033 and Florida 07 × GP-NC WS 16 (SPT06-06) (Tallury et al., 2014). Eleven diploid *Arachis* species, three induced allotetraploid interspecific hybrids (all 2n = 4x = 40): *A. batizocoi* Krapov. & W.C. Greg. × *A. stenosperma*; *A. gregoryii* C.E. Simpson, Krapov. & Valls × *A. stenosperma*, and *A. duranensis* × *A. ipaensis* were also included. Supplemental Table 9 shows all 384 samples assayed. DNAs for tetraploid cultivars and accessions were extracted from leaves of greenhouse-grown plants or seed or hypocotyl and roots from 7-day-old seedlings. DNAs from RIL populations were extracted from a pool of 15–20 leaflets harvested from F_6_ plots grown in the field in Tifton, GA. DNA from diploid species was extracted from leaf tissue. All DNAs were extracted using a QIAGEN DNAeasy Plant mini kit and quantified using picogreen.

### Identification of Markers that Reveal Tetrasomic Recombination

All tetraploid lines genotyped on the array were analyzed using Axiom Analysis Suite v.1.1.0.616 using default polyploid threshold configurations. All SNPs that were placed into the categories PolyHighResolution, NoMinorHom, OTV, and Other were further curated manually. Markers where more than one third of the genotypes were scored as heterozygous were evaluated first. Next, the clustering for unexpected clusters using criteria similar to Leal-Bertioli et al. (2015a) was assessed. These markers with excessive heterozygosity and unexpected clusters were then determined to be tetrasomic, and the genotypes in the unexpected clusters were scored as tetrasomic. Supplemental Table 4 shows all 1193 tetrasomic markers and the scores for the 175 tetraploid genotypes including the USDA mini core collection and the 63 cultivars and breeding lines.

### Signatures of Selection

Using an updated pedigree of runner market-type cultivars (Supplemental Figure 4) first published in Isleib et al. (2001), 42 combinations of parents and progeny selections, which we refer to as trios, were identified. If the parent genotype was not assayed, data from the grandparents were used if all available alleles were accounted for. For example, Florispan was a progeny of GA207-3 and Early Runner. Neither of these genotypes was available to include on the array, but all four grandparents (Dixie Giant, Spanish 18-38, Basse, and Small White Spanish) were available. All alleles were selected from those four genotypes. Using custom scripts, every trio for each marker was tested (Supplemental File 1). If for a trio, a marker was polymorphic in the parents, the selected allele in the progeny was counted as one test. The test was a binomial exact test for segregation distortion as in Nixon (2006) with the null hypothesis of random selection and a 1:1 allele ratio. Analysis was carried out in R (v 3.2.3; 2016) using the binom.test function with a probability of 0.5 and an alternative model of greater than expected. *P* values were corrected for multiple testing using a Benjamini-Hochberg correction to test for a false discovery rate of 0.1.

### Tracking Ancestor Alleles

Seven ancestors contributed all of the marker polymorphisms in runner-type cultivars grown in the southeastern United States: Basse, Spanish 18-38, Dixie Giant, Small White Spanish, Jenkins Jumbo, Virginia Bunch 67, and PI203396. All of the marker polymorphisms from these ancestors were present in the 18 modern cultivars assayed. This gave us the ability to trace polymorphisms specific to each ancestor. Specific marker polymorphisms from each ancestor were identified by selecting those where all other ancestors were fixed for the opposite nucleotide. Markers where any of the ancestors had a heterozygous call were discounted. Tetrasomic genotype scores on chromosome A04 from PI203396 were also disregarded because they were only retained in Tifrunner. Using custom scripts, marker polymorphisms from each ancestor in the 18 cultivars were tracked, and the percentage of available unique alleles for each breeding cycle was calculated (Supplemental Table 8).

### Tracking Recombination

Eleven breeding paths were assembled when genotyping information for at least one parent at each cross was available. One parent was enough to determine if there was a phase change in the selected progeny between two markers. The real opportunity for a phase change by knowing which markers were polymorphic between the two parents was not taken into account. This is because, in a breeding situation, the actual alleles of the parents being crossed are not known. The process measures the actual recombination occurring after crossing and selection when breeders are only making crossing decisions based on the phenotypes of the parents. Markers were tested for each path in overlapping pairs ordered based on their putative position relative to the *A. duranensis* and *A. ipaensis* pseudomolecules. Changes of phase between two markers were recorded at each crossing cycle within the specific path. The crossing cycles here do not correspond to the pedigree cycles but instead to crossing cycles within the breeding path. For example, in one path originating with the cross Dixie Giant × Small White Spanish, Southern Runner is the progeny of the fourth cross from the original and so is assigned to cycle four. In another path originating with the cross PI203396 × Florunner, however, Southern Runner is assigned to cycle one or the progeny of the first cross from the ancestor. Wilcox rank sum tests were carried out in R v.3.2.3 using the wilcox.test() function.

## Funding

This project was funded by the Feed the Future Innovation Lab for Collaborative Research on Peanut Productivity and Mycotoxin Control (Peanut and Mycotoxin Innovation Lab), supported by funding from the United States Agency for International Development (USAID) (P.O.-A., S.A.J., D.J.B., R.K.V., M.B.); the US-Israel Binational Agricultural R&D Fund (BARD) project, IS-4540-12 (P.O.-A., R.H., S.A.J.); the Agriculture and Food Research Initiative competitive grant 2012-85117-19435 of the USDA National Institute of Food and Agriculture (P.O.-A., C.C.H.); the Peanut Foundation (P.O.-A., S.A.J., R.K.V.), and Bill & Melinda Gates Foundation (grant no. OPP1114827) funded Tropical Legumes III project (R.K.V.).

## Author Contributions

P.O.-A., R.K.V., and S.A.J. conceptualized the research; Y.C., C.C., D.J.B., S.C.M.L.-B., J.V., N.A.B., R.H., M.B., T.G.I., and C.C.H. provided genetic resources and data; J.C., Y.C., C.C., G.A., M.K.P., S.N.N., A.C., D.J.B., S.C.M.L.-B., and B.A. performed experiments, conducted data analysis, and curated data; J.C. and J.V. contributed to the methodology; J.C. wrote the original draft and was responsible for data visualization; P.O.-A., S.A.J., R.K.V., Y.C., M.K.P., D.J.B., S.C.M.L.-B., C.C., and J.C. revised the manuscript; P.O.-A. administered the project.

## Figures and Tables

**Table 1:** Putative Peanut Orthologs of Soybean Maturity Genes, Meristem Identity Genes, and Known Peanut FAD2 Genes.

Gene	Organism	Peanut ortholog	Chr	Position	Significance	Nearest significant PHS	PHS	Average cycle polymorphic
*E1*	Soybean	Araip.W7PF8	B09	105 492 707...105 493 192	Binomial exact + simulation + PHS	110 532 140	42.11	4.58
*E2*	Soybean	Aradu.V81ZJ	A05	108 493 627...108 500 291	N/A			
		Aradu.61FJ2	A09	117 706 867...117 717 279	Odds			
		Araip.WW4C8	B09	138 395 951...138 406 437	Binomial exact + simulation + PHS	137 665 621	61.23	3.85
*DT1*	Soybean	Araip.T6XJY	B08	5 521 956...5 523 342	Binomial exact + simulation			5.00
		Aradu.RJP5K	A08	28 292 902...28 294 197	Binomial exact + simulation			3.92
*E3*	Soybean	Aradu.E3ZED	A06	6 070 225...6 074 408	N/A			
		Araip.HY5UP	B06	10 971 497...10 975 711	N/A			
*E4*	Soybean	Araip.K62H2	B09	26 116 014...26 122 370	Binomial exact + simulation			3.18
*LFY*	Arabidopsis	Aradu.BZU3P	A08	47 812 969...47 816 223	Binomial exact + simulation + PHS	48 109 634	21.28	4.67
		Araip.T09RD	B08	128 285 886...128 289 162	Binomial exact + simulation			3.86
*FAD2*	Peanut	Aradu.G1YNF	A09	114 690 776...114 693 267	Odds	113 715 476	50.76	3.75
		Araip.65EGG	B09	141 478 208...141 479 692	Binomial exact + simulation + PHS	142 124 962	33.91	5.31

Blastx was used to identity putative orthologs of E1, E2, E3, E4, DT1, and LFY. For each gene, the physical position in the *A. duranensis* and *A. ipaensis* genome sequences, if the nearest marker was significant and by which test, the nearest significant marker with an outlier PHS value, and the average cycle that marker was still polymorphic are presented.

## Supplemental Information

Document S1. Supplemental Results, Supplemental Methods, and Supplemental Figures 1–8

Supplemental Table 1. Final Array Design

Supplemental Table 2. Re-sequencing Stats for 20 Tetraploid *A. hypogaea* Genotypes

Supplemental Table 3. Stats for Sequence Reads for Diploid SNP Identification

Supplemental Table 4. Genotype Calls for Tetrasomic Markers in All Tetraploid *A. hypogaea* Genotypes Assayed, Related to Figure 3 and Figure 4

Supplemental Table 5. Simulated Thresholds for Selection due to Genetic Drift at *p* < 0.01

Supplemental Table 6. Loci Showing Directed Selection

Supplemental Table 7. GO Enrichment in Selected Loci

Supplemental Table 8. Tracking Ancestor-Specific Alleles through Eight Cycles of Breeding in US Runner Cultivars

Supplemental Table 9. 384 Accessions Genotyped on the Array

Supplemental File 1. Scripts Used in Custom Analyses
