# Supplementary material for: Genome-wide SNP Genotyping Resolves Signatures of Selection and Tetrasomic Recombination in Peanut
Source: Mol Plant. 2017 Feb 13;10(2):309–22. doi: 10.1016/j.molp.2016.11.015 (PMC5315502; doi:10.1016/j.molp.2016.11.015)
Supplement: Document S1. Supplemental Results, Supplemental Methods, and Supplemental Figures 1–8 [file mmc1.pdf]

Supplemental Information

Supplementary Results

Tetraploid markers

The array was evaluated using 384 tetraploid and diploid genotypes, including the genotypes used to design the array, the USDA mini core collection, 96 individuals from each of two RIL populations, a selection of US released cultivars spanning the history of US peanut breeding, ten diploid species, four induced allotetraploid individuals, and an *A. monticola* accession (Table S9). Evaluating the tetraploid genotypes, a total of 15,287 markers produced scorable, polymorphic markers. The markers fall into the following categories (Figure S7): 4,647 PolyHighResolution (24.18%; genotypes forming three clusters within expected priors) (Figure S7A); 9,818 NoMinorHom (65.25%; no representative genotype with homozygous minor allele) (Figure S7B); 1,330 Other (8.84%; falling below two or more thresholds) (Figure S7C); 151 OTV (1%) (Figure S7D; off target variants); and 109 CallRateBelowThreshold (0.87%; genotype call rate is below threshold) (Figure S7E).

Called SNPs were filtered from the WGS sequence data using the pipeline SWEEP (Clevenger and Ozias-Akins, 2015). The array provided an opportunity for large scale validation of the pipeline using WGS *A. hypogaea* data. Out of the 42,658 putative markers identified from the WGS data, 14,223 (33%) were scorable, polymorphic markers. The other polymorphic markers (1,064) were identified from diploid species.

Interspecific markers

Cultivated peanut has undergone a genetic bottleneck and interspecific populations are important tools to introduce beneficial alleles from diploid wild relatives. Interspecific populations have been made using two methods, known as the hexaploid and tetraploid routes (Stalker *et al.*, 1979; Simpson 1993). The hexaploid route first generates a triploid hybrid from a cross between the tetraploid *A. hypogaea* and a diploid species, the resulting hybrid is then colchicine treated to create a hexaploid plant with low fertility that is then selfed through multiple generations and by spontaneous loss reaches a tetraploid state. The tetraploid route

begins by hybridization of A- and B-genome species, and then making the hybrid compatible with tetraploid *A. hypogaea* through chromosome doubling to recover a synthetic allotetraploid. Markers that distinguish diploid species are extremely important for mapping and identifying beneficial alleles from these populations. With this in mind, 13,732 putative SNP markers polymorphic between six diploid species were included. Ten diploid species were analyzed on the array including *A. duranensis* V14167 (A), *A. duranensis* K7988, *A. stenosperma* V10309 (A), *A. cardenasii* GKP10017 (A), *A. villosa* Benth. V12812 (A), *A. correntina* (Burkart) Krapov. & W.C. Greg. V12812 (A), *A. ipaensis* K30076 (B), *A. magna* K30097 (B), *A. gregoryii* V6389 (B), and *A. batizocoi* K9484 (K but B-compatible; Leal-Bertioli *et al*., 2015b). Additionally, four induced allotetraploid interspecific hybrids were analyzed on the array, including an *A. batizocoi x A. stenosperma*, an *A. gregoryii x A. stenosperma*, a first generation *A. duranensis x A. ipaensis* induced allotetraploid, and an *A. duranensis x A. ipaensis* induced allotetraploid after nine self-pollinations.

Analysis of these diploids and interspecific hybrids resulted in 53,135 (93.7%) polymorphic markers; 22,221 PolyHighResolution (38.2%), 3,669 NoMinorHom (6.3%), 12,823 Other (22%), 9,760 OTV (16.8%), and 4,662 CallRateBelowThreshold (8%). Polymorphism within A- or B-genome species (Figure S8) shows that the array will be useful for genotyping interspecific populations. Polymorphism between *A. duranensis* and the other A-genome species assayed ranges from 8,149 to 11,044 polymorphic markers, and between *A. ipaensis* and the B genome-compatible species, from 6,553 to 19,400 polymorphic markers were detected. Polymorphism between *A. duranensis* V14167 and *A. duranensis* K7988 includes an additional 3,600 intraspecific polymorphic markers.

Three induced allotetraploids derived from diploid hybrids were genotyped on the array to test the efficacy for genotyping interspecific populations (Figure 5B). Polymorphic markers between these hybrids and three elite cultivars (Florunner, Tifguard, and Georgia-06G) that could potentially be used as *A. hypogaea* backcross recurrent parents averaged 29,748 polymorphic markers between the cultivars and the *A. batizocoi* x *A. stenosperma* hybrid, 9,924 polymorphic markers for the *A. ipaensis* x *A. duranensis* hybrid, and 25,238 polymorphic markers for the *A. gregoryii* x *A. stenosperma* hybrid.

Supplementary Experimental Procedures

Filtering, Selection, and Formatting of SNPs for the array

SWEEP-filtered SNPs were filtered to be within 10 kb of *A. duranensis* and *A. ipaensis* annotated genes (Bertioli *et al*., 2016). Sequences for each SNP were extracted using custom scripts in the format prescribed by Affymetrix: (1) 35 bp surrounding each SNP with (2) the two polymorphic bases ordered alphabetically. Selected SNPs were prioritized if enough Illumina read data existed to assemble the cultivated sequence for each SNP. Extracted sequences were then filtered for single copy loci by BLAST against the *A. duranensis* and *A. ipaensis* pseudomolecules and only selecting those that had a unique hit of ≥ 94% identity or across at least 60 aligned bases within each sub genome separately. This stringent filtering resulted in 113,787 SNPs sent to Affymetrix for selection.

Identification of diploid SNPs

RNA sequencing data from *A. stenosperma* Krapov. & W.C. Greg. and *A. cardenasii* Krapov. & W.C. Greg. were mapped to the *A. duranensis* pseudomolecules (Bertioli *et al*., 2016; peanutbase.org). RNA sequencing data from *A. batizocoi* and genomic sequencing data from *A. magna* were mapped onto the *A. ipaensis* assembled pseudomolecules (Bertioli *et al*., 2016; peanutbase.org). Mapping was performed by using BWA mem v. 0.7.10 with default parameters (Li and Durbin, 2009). Additionally, three accessions of *A. duranensis* (PI475845, ICG8123, and ICG8238; Pandey *et al*., 2017) were used to call SNPs within species. SNPs were called using Samtools v0.1.9 and filtered as follows: (1) At least 4 reads with the alternative base and no reference bases in the genotype showing the SNP (2) no SNPs within 35 bp of each other and (3) within 10kb of annotated gene models. SNPs randomly selected (5,000) from each diploid species representing 500 SNPs from each chromosome for each species were chosen. These 25,000 SNPs were added to the 113,787 tetraploid SNPs provided to Affymetrix for selection. Table S2 and S3 shows read statistics for each species.

Simulation of neutral model tracking fixation across breeding cycles

Because breeding and selection uses small sample sizes, alleles can be rapidly fixed due to genetic drift and not as a result of intensive selection. A simulation was carried out assuming neutral selection using the pedigree as a guide. At each cycle, the breeding process was recreated using the parental genotypes and 50 released progeny were generated. For each cross, the distribution of observed distances between recombination (see Figure S1) was sampled and each parent's alleles were transferred to the progeny based on random independent assortment. Each cycle was recreated based on the actual pedigree. Briefly, the first cycle was generated from 25 released progeny derived from the cross of Basse x Spanish 18-38 and 25 progeny generated from the cross of Dixie Giant x Small White Spanish. For cycle 2, 50 released progeny were generated from randomly mated pairs of cycle 1 progeny. Cycle 3 progeny were generated from randomly mated pairs of cycle 2 progeny and 25% of the time one parent was Jenkins Jumbo, as at cycle 3 Jenkins Jumbo was included as a parent. Cycle 4 introduced as parents Virginia Bunch 67 and PI203396. PI295785 was introduced as a parent in cycle 5 and PI203396 was used as a parent for this cycle as well. For cycles 6, 7, and 8, progeny were generated from randomly mated pairs of the previous cycle. The percent of fixed alleles across all markers for all 50 generated progeny was calculated for each cycle. This simulation was carried out 10,000 times and a second time for 5,000 times. The average, standard deviation, and 99% percentile of the distribution was calculated. The simulation python script is available in Supplementary file S1 as simulate_allele_fixation.py

Neutral selection/drift simulation to derive a null distribution

To account for the fixation of alleles in the absence of selection due to genetic drift and small effective population sizes, simulations were performed under a neutral model by recreating pedigree selection of an inbred crop with different F_2 starting population sizes and different selection intensities. Population sizes of 200, 300, and 400 individual F_2 families based on the number of seeds available from a single F_1 plant from a specific cross. Selection intensities of 0.1, 0.2, and 0.3 were used for all population sizes. The simulations were performed for each marker, genome-wide, to account for marker-specific allele frequencies within the germplasm sampled. Fifty genotypes were randomly selected for mating. Of those fifty pairs, if a pair is polymorphic for the marker of interest, pedigree selection is carried out under neutral selection

and the selected allele is noted. Segregation distortion is not taken into account and segregation ratios used are those expected in a self-pollinated crop. Random selection at each generation is carried out based on the selection intensity until a homozygous allele is randomly selected. The random crosses sampled from the actual data give for each simulation a different number of tests for possible selection given the real allele frequencies present. The simulation was carried out for each marker 100 times for a total of 553,700 simulations per starting population size and selection intensity. Nine combinations of population size and selection intensity were carried out for a total of 4,983,300 simulations. For each number of tests up to 45, the 99th percentile was taken as a threshold for significance of directed selection. The average across all combinations was taken as a threshold for significance. These results are shown in Table S5. There is not sufficient power to detect selection under this model until nine tests have been performed. From nine to twenty tests the threshold is selection in more than 80% of polymorphic crosses. The simulations provide a very stringent threshold to test for selection. The simulation python script is available in Supplementary file S1 as simulate_neutral_selection.py

Calculation of Pairwise Haplotype Sharing (PHS)

Pairwise Haplotype Sharing (PHS) for each marker in the two populations of cultivars released in cycles 4, 5, and 6 and cultivars released in cycles 7, and 8 was compared against the PHS among ancestor/founder lines. PHS was calculated as in (Toomajian *et al*., 2006) as

$$PHS_{x_A} = \frac{\sum_{i=1}^{p-1} \sum_{j=i+1}^{p} Z_{ijx}}{\binom{p}{2}} - \frac{\sum_{i=1}^{n-1} \sum_{j=i+1}^{n} Z_{ijx}}{\binom{n}{2}}$$

and

$$Z_{ijx} = \frac{d_{ijx} - \bar{d}_{ij}}{\sigma_{ij}}$$

with a slight adjustment. This statistic has low power for haplotypes that have been fixed in a given population, and since the number of released peanut cultivars is low, we adjusted the PHS by the ancestor population instead of within populations. This way the extent of increased haplotype sharing in each population was compared to the extent of haplotype sharing present within the narrow starting genetic base. d_{ijx} is the physical distance according to the diploid

pseudomolecules over which individuals *i* and *j* are identical around position *x,* $\bar{d}_{ij}$ is the mean of the distance the individuals are identical genome-wide, σ_{ij} is the standard deviation of the genome wide distribution, and individuals *i* and *j* share the same allele at position *x.* Then, p, is the number of individuals that share the same allele at position x and n is the number of ancestor lines. PHS was calculated for each allele at every marker and then the largest value was taken. Values greater than the 99th percentile of the genome wide distribution of PHS within each population were determined to be significant. For cycles 4, 5, and 6 this value was 8.11 and for cycles 7 and 8 it was 9.68. The python script to calculate PHS is included in Supplementary file S1 as calculate_pairwise_haplotype_share.py.

GO enrichment with selected loci

The sum of each GO term present in the *A. duranensis* and *A. ipaensis* genome sequences (peanutbase.org) and within each selected locus was counted. A hypergeometric test for enrichment testing the distribution of GO terms within the locus compared to the genome-wide distribution was then used using the R function phyper() and each p-value was adjusted for multiple testing using a Benjaminni-Hochberg correction. Adjusted p-values were further filtered to be less than 0.001 to control for false positives due to smaller sample size. Loci with no GO terms with more than 3 genes represented were not considered further due to uncertainty.

Frequency of shared haplotypes

The mini core population was used as an estimate of haplotype frequency in *Arachis hypogaea*. All possible 20 marker haplotypes at 5 marker intervals were identified in the 111 mini core genotypes and were ranked for their frequency. The top eight haplotypes were then assessed for their frequency in the ancestor/founder genotypes and the two populations comprising of cultivars released in cycles 4,5, and 6 and cultivars released in cycles 7, and 8 with a unique color based on their mini core frequency. Because PI203396 had such a large effect on peanut breeding and production, contributing TSWV resistance/tolerance to the cultivated germplasm, it's haplotype was given a color as well. In addition, all other haplotypes with frequencies less than the top eight were given a color. These haplotype frequencies were

graphed for each population with the population-specific haplotype frequency on the y-axis and the marker position in five marker intervals on the x-axis. The number of unique haplotypes per marker position and the frequency of unique haplotypes relative to population number was derived from the same analysis. The associated python script is available in Supplementary file S1 as haplotype_frequency.py.

Haplotype diversity analysis

The haplotype diversity was calculated as pairwise diversity, π, in 20 marker haplotypes moved in five marker intervals across each chromosome within each population. The populations were the 11 mini core genotypes representing an estimate of the available genetic diversity within *Arachis hypogaea*, the ancestor/founding genotypes, the cultivars released in cycles 4,5, and 6, and the cultivars released in cycles 7, and 8. The cultivars released in cycles 1,2, and 3 were not assessed because these cultivars represented germplasm from only two crosses. Additional ancestor/founder alleles were introduced starting in cycle 4. In addition cycles 4,5, and 6 represent cultivars released with Florunner as a common parent among them and cycle 7, and 8 represent cultivars further removed from Florunner and cultivars that represent the move to high oleic acid content. Diversity was represented as the number of pairwise differences per marker across each haplotype window. For each population, π was compared to the estimated possible diversity as $\log_2(\pi_p/ \pi_m)$ where π_p represents the population of interest and π_m represents the mini core collection. Therefore, loss of diversity results in a negative value.

Supplemental Figures

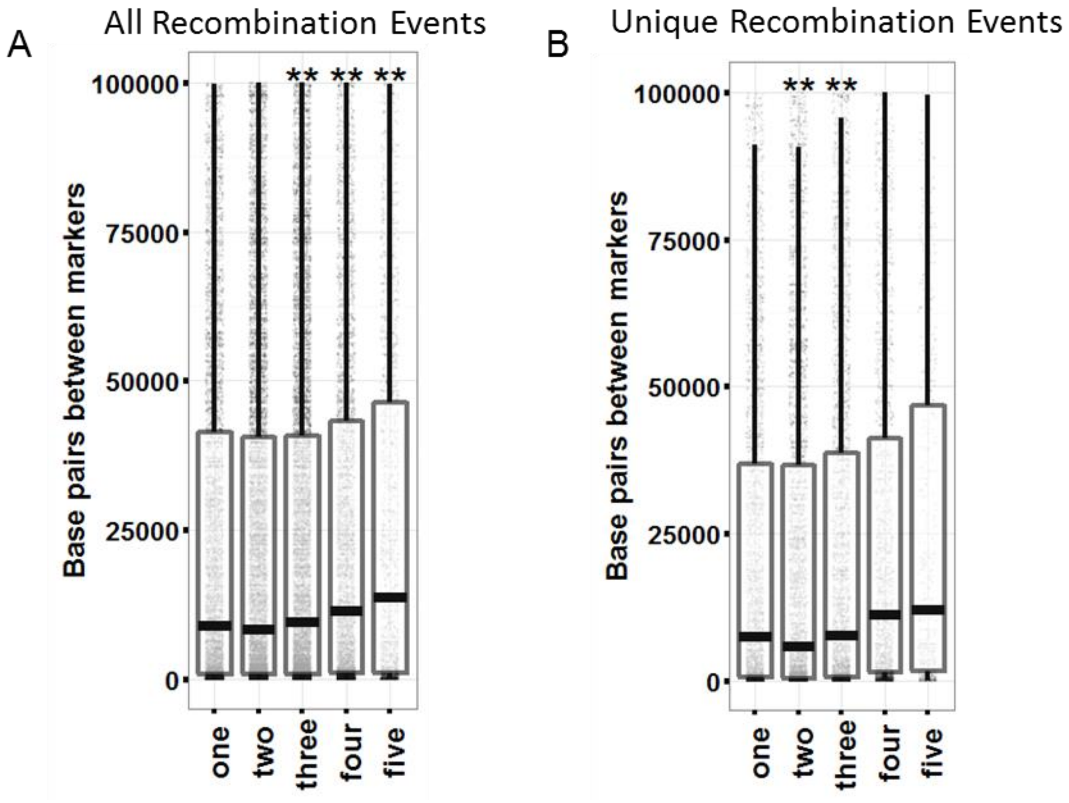

Figure S1: Distance between markers that show a phase change (recombination) after each number of crosses, inbreeding, and cultivar selection, related to Figure 7A. A) all phase changes. B) phase changes specific to each cross number. Asterisks indicate significant difference in distribution between the cross number and the next cross by Wilcox Signed-Rank Test. There is a decrease in gain in breaking up large linkage blocks after three crosses as distance between markers showing a phase change increases even for unique events.

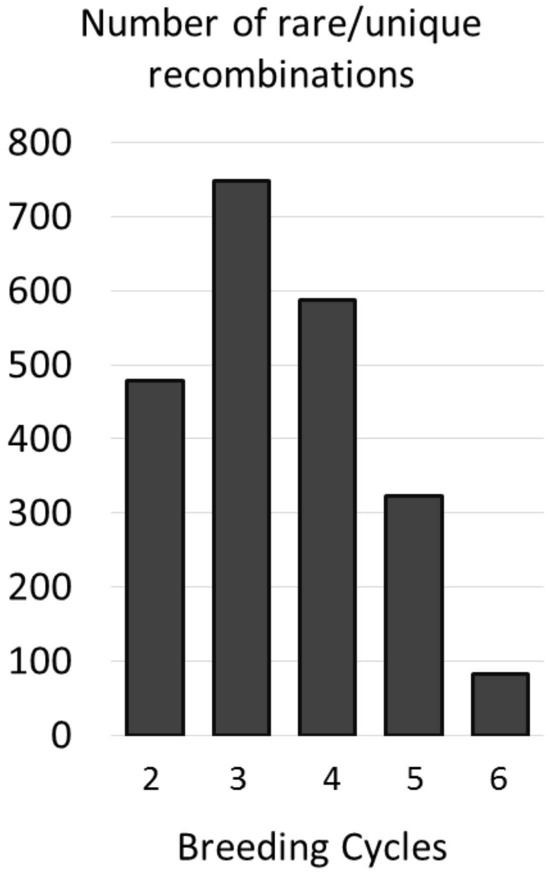

Figure S2: Unique/rare recombinations per cycle, related to Figure 7A. Average number of recombination events specific to a breeding cycle out of ten parent-progeny breeding paths where phase changes between markers could be determined.

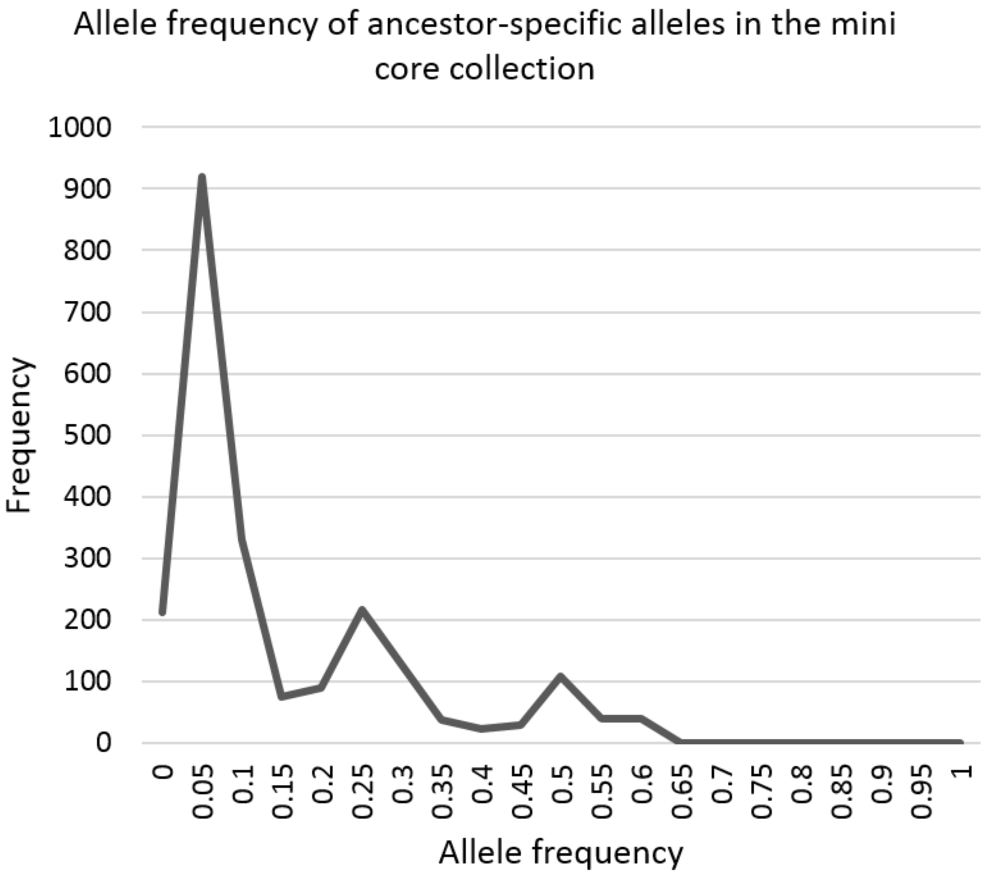

Figure S3: Frequency of ancestor-specific alleles in the mini core collection.

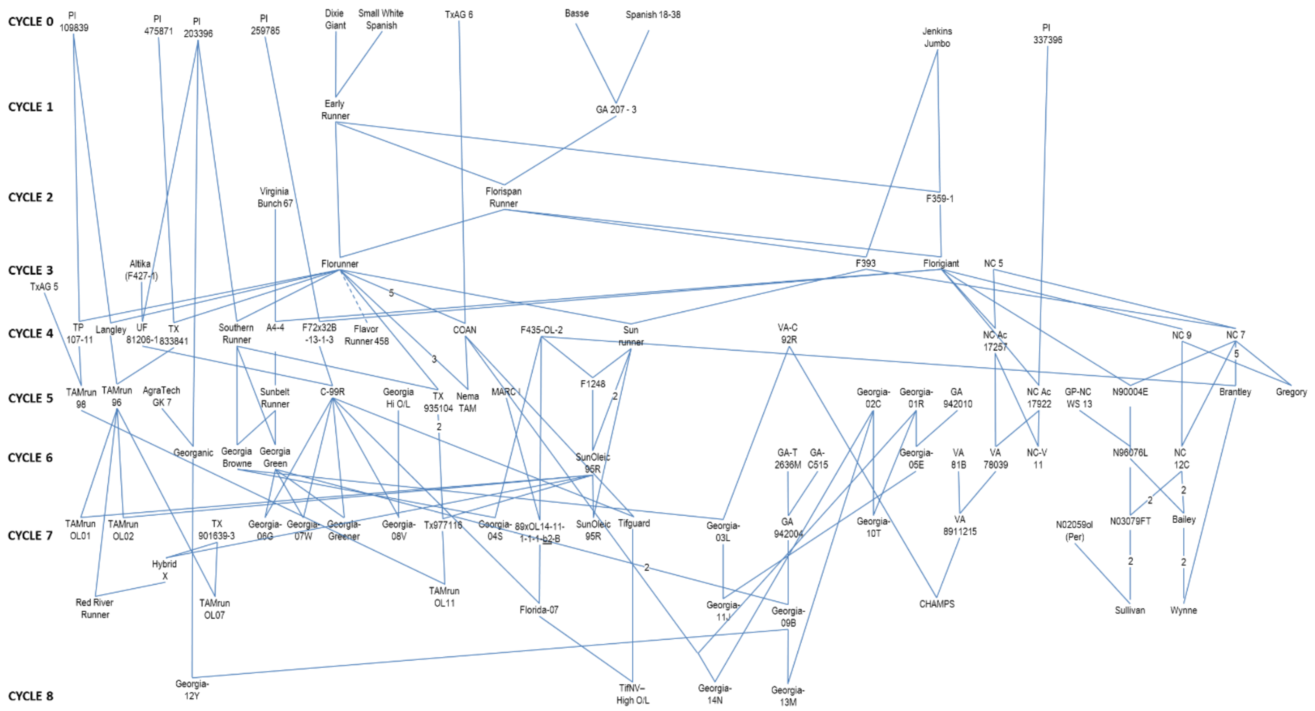

Figure S4: Pedigree of US runner cultivars. Each cultivar is assigned to a cycle based on Isleib *et al.*, 2000, where a cycle is the number of crosses away from the original ancestors. Pedigree also available on peanutbase.org/pedigree2000.

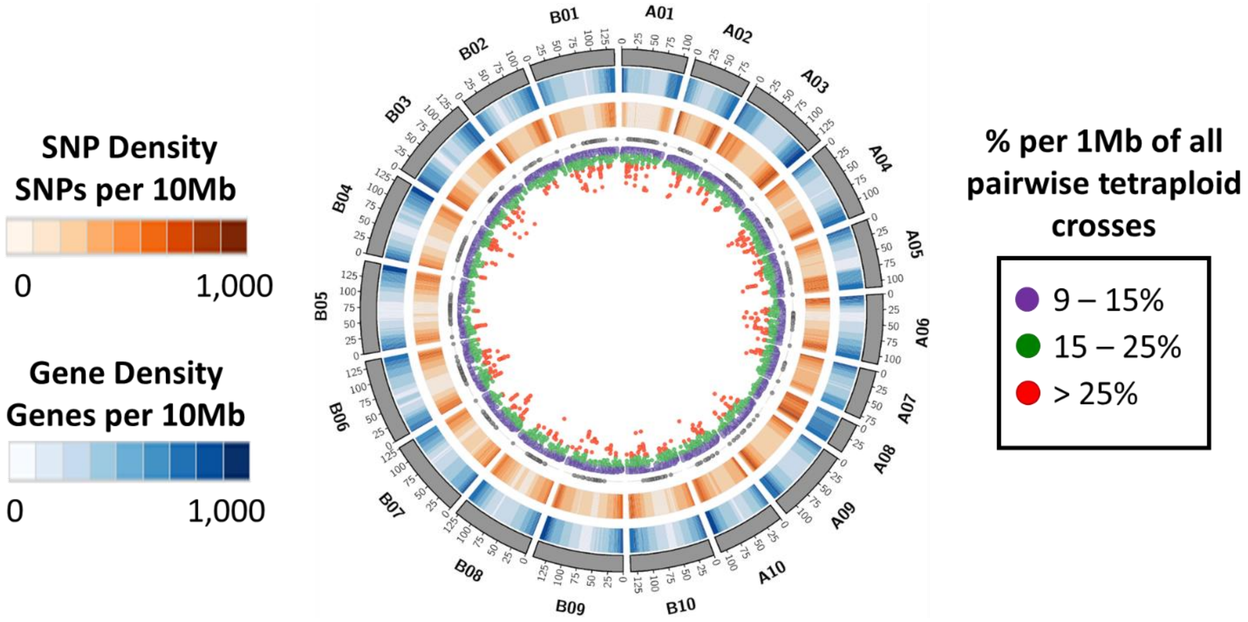

Figure S6: Final array design SNP density and predicted polymorphism. Circos plot showing *A. hypogaea* genome represented by *A. duranensis* and *A. ipaensis* pseudomolecules. Outer ring is gene density of annotated predicted genes. Second ring is SNP density of the 58,233 SNPs in the final design of the Axiom_Arachis array. Scatter plot shows predicted polymorphism among the 21 4x genotypes used to design the array. Percentage polymorphic is all pairwise crosses. Scatterplot is sliding window of 1Mb windows sliding 500kb. Gene density and SNP density are sliding windows of 10 MB sliding 5Mb.

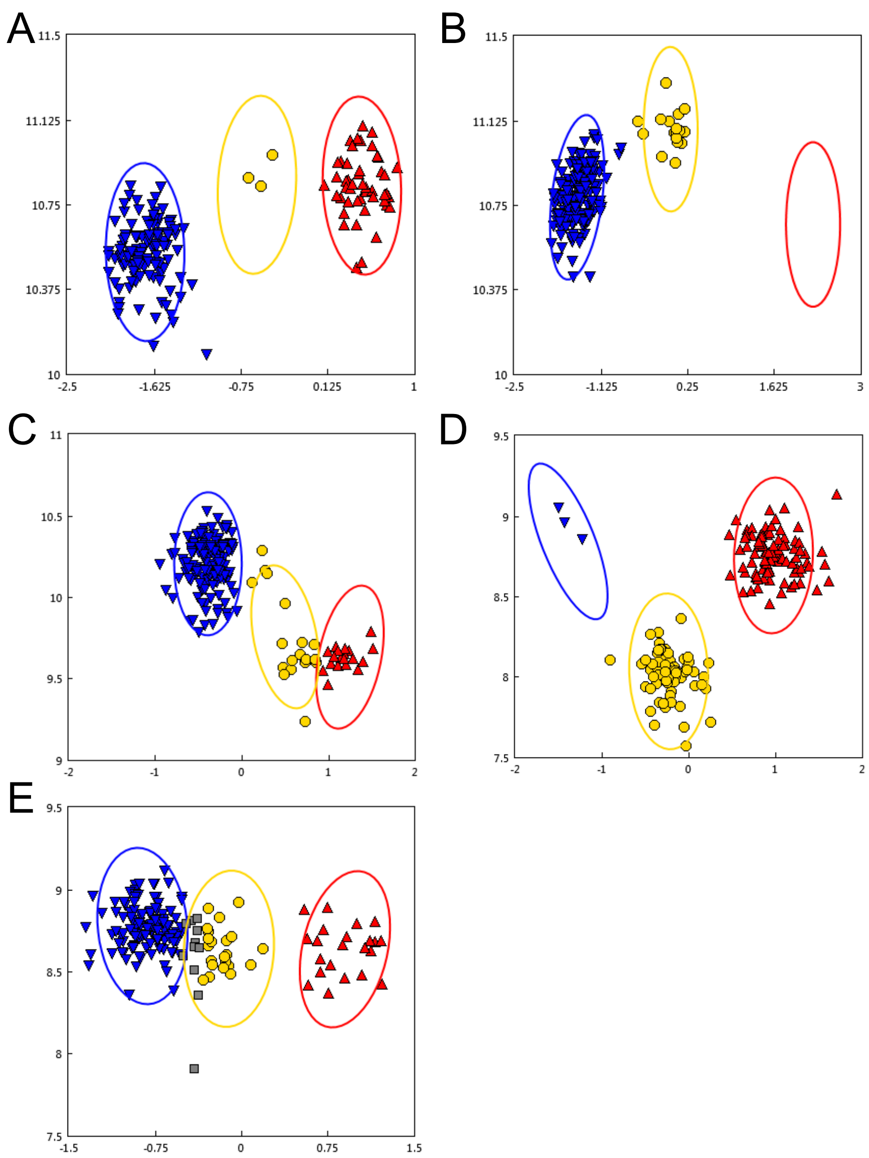

Figure S7: Examples of 5 different categories of markers segregating in tetraploid germplasm. (A) PolyHighResolution; genotypes forming three clusters within expected priors (B) NoMinorHom; no representative genotype with homozygous minor allele (C) Other; falling below two or more thresholds (D) OTV; Off target variant (E) CallRateBelowThreshold; genotype call rate is below threshold

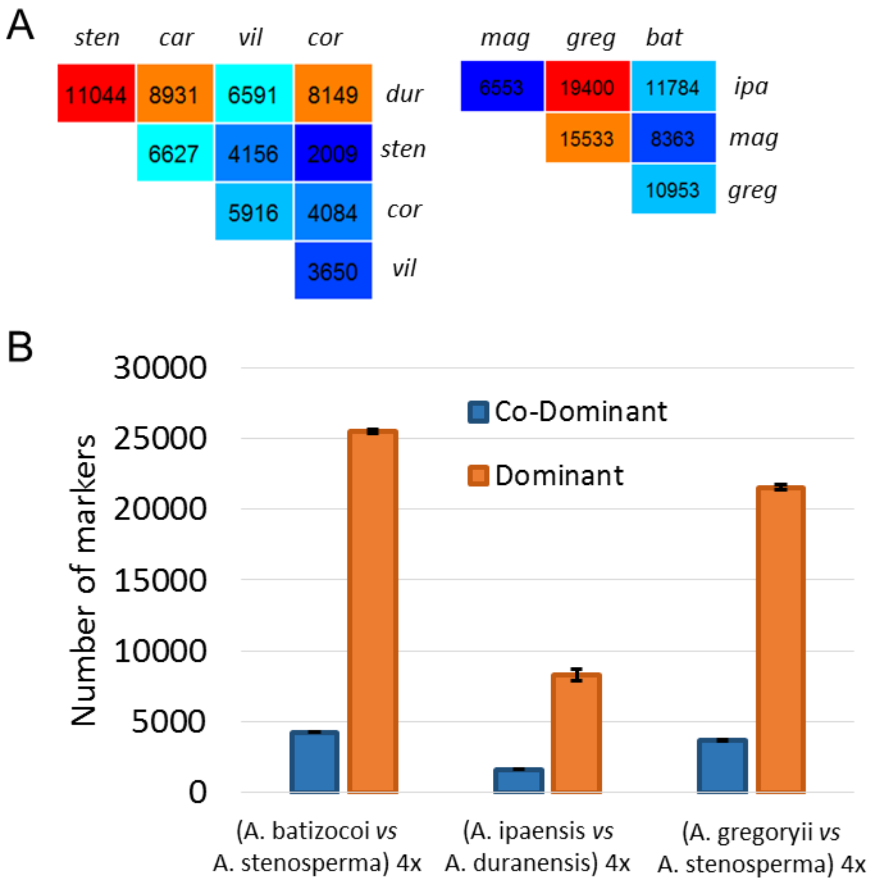

Figure S8: Polymorphism within wild diploid *Arachis* species and between interspecific hybrids and elite cultivated genotypes. A) polymorphic markers between A genome (left) and B genome (right) compatible species; B) average number of polymorphic markers between three interspecific allotetraploid hybrids and elite parents Florunner, Tifguard, and Georgia-06G. Sten is *A. stenosperma*; car is *A. cardenasii*; vil is *A. villosa*; cor is *A. correntina*; mag is *A. magna*; greg is *A. gregoryii*; bat is *A. batizocoi*; dur is *A. duranensis*; ipa is *A. ipaensis*

Supplementary References

Li, H., Durbin, R. (2009). Fast and accurate short read alignment with Burrows-Wheeler Transform. Bioinformatics 25:1754-1760.

Pandey, M., Agarwal, G, Kale ,SM, Clevenger, J, Nayak, SN, Sriswathi, M, Chitikineni, A, Chavarro, C, Chen, X, Upadhyaya, HD, Vishwakarma, MK, Leal-Bertioli, S, Liang, X, Bertioli, DJ, Guo, B, Jackson, SA, Ozias-Akins, P, Varshney, RK. (2017). Development and Evaluation of a High Density Genotyping 'Axiom_Arachis' Array with 58 K SNPs for Accelerating Genetics and Breeding in Groundnut. Sci Rep 7: doi: 10.1038/srep40577.
